# Decade of 2D-materials-based RRAM devices: a review

**DOI:** 10.1080/14686996.2020.1730236

**Published:** 2020-03-18

**Authors:** Muhammad Muqeet Rehman, Hafiz Mohammad Mutee Ur Rehman, Jahan Zeb Gul, Woo Young Kim, Khasan S Karimov, Nisar Ahmed

**Affiliations:** a Faculty of Electrical Engineering, Ghulam Ishaq Khan Institute of Engineering Sciences and Technology, Topi, Pakistan; b Department of Mechatronics & Biomedical Engineering, AIR University, Islamabad, Pakistan; c Faculty of Electronic Engineering, Jeju National University, Jeju, South Korea

**Keywords:** 2D materials, resistive switching, nonvolatile, bipolar & unipolar, RRAMs, fabrication technology, planar & sandwiched structure, 105 Low-Dimension (1D/2D) materials, 201 Electronics / Semiconductor / TCOs, 306 Thin film / Coatings, 503 TEM, STEM, SEM, Memory devices, RRAMs

## Abstract

Two dimensional (2D) materials have offered unique electrical, chemical, mechanical and physical properties over the past decade owing to their ultrathin, flexible, and multilayer structure. These layered materials are being used in numerous electronic devices for various applications, and this review will specifically focus on the resistive random access memories (RRAMs) based on 2D materials and their nanocomposites. This study presents the device structures, conduction mechanisms, resistive switching properties, fabrication technologies, challenges and future aspects of 2D-materials-based RRAMs. Graphene, derivatives of graphene and MoS_2_ have been the major contributors among 2D materials for the application of RRAMs; however, other members of this family such as hBN, MoSe_2_, WS_2_ and WSe_2_ have also been inspected more recently as the functional materials of nonvolatile RRAM devices. Conduction in these devices is usually dominated by either the penetration of metallic ions or migration of intrinsic species. Most prominent advantages offered by RRAM devices based on 2D materials include fast switching speed (<10 ns), less power losses (10 pJ), lower threshold voltage (<1 V) long retention time (>10 years), high electrical endurance (>10^8^ voltage cycles) and extended mechanical robustness (500 bending cycles). Resistive switching properties of 2D materials have been further enhanced by blending them with metallic nanoparticles, organic polymers and inorganic semiconductors in various forms.

## GRAPHICAL ABSTRACT

**Figure:**
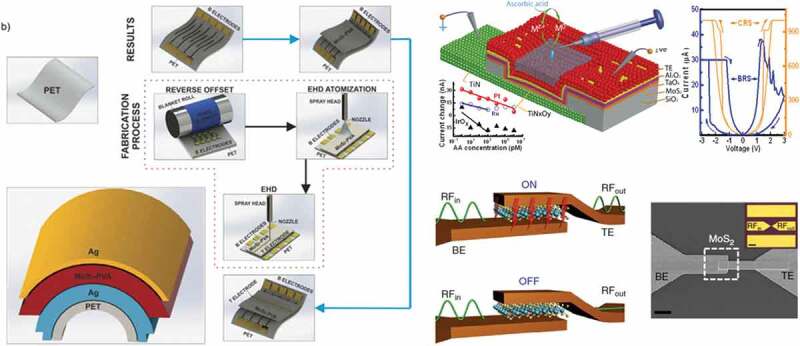

## 1. Introduction

Flash memory devices based on silicon have ruled the electronic industry due to their successful and distinguished nonvolatile memory (NVM) characteristics [1–3]. Nevertheless, existing NVM devices including flash memories face a few severe problems in their operation of charge storage such as slow programming speed, relatively high operating voltage, and poor electrical endurance [4]. Moreover, their endless shrinking (to enhance the data storage capacity) is anticipated soon to reach its basic scaling limit mainly due to the difficulty of charge retention in dwindling dimensions [5]. Typical memory devices have served the needs of data storage for several years but are no longer a solution to ever increasing demand for high-density memory storage. Information storage technology based on silicon is facing physical and theoretical limitations. Therefore, the challenge of exploring and developing new techniques and devices exist to overcome data storage deficiency. A comprehensive study in this regard for the various available advanced memory technologies has been reported [6].

As a substitute to the typical silicon-based memory, resistive random access memory (RRAM) devices have emerged as the potential candidate for the future flexible NVM device owing to their standout features such as simple structure [7], low power consumption [8], high scalability [9], low cost (≈0.019 $ GB^–1^) [6], enormous data storage (5.62 × 10^10^ bits cm^–2^) [6], highly desirable multi-bit data storage/unit cell, compatibility with CMOS technology [10,11], and compatibility with fabrication techniques [12,13]. Intermediary resistive states can also be obtained that give rise to added functionalities such as neuromorphic processing, filters, logic, arithmetic and multibit storage. Owing to these promising attributes, 47 billion USD has been invested in these memory devices in 2016 [6]. Furthermore, RRAM devices offer flexibility unlike hard disks and flash drives that seems to be the future of electronic industry.

NAND Flash is the most used memory these days with a major disadvantage of current leakage when it reaches the nanometer scale to achieve high density, mainly due to the integration of capacitor in it. Current leakage can result in loss of information [14] therefore, resistive memory devices are superior to capacitive memory devices due to high scalability without compromising data storage. Some of the most remarkable RRAM devices reported till now include the RRAMs with a superfast switching speed (300 ps) [15–18], extremely low power losses (0.1 pJ) [15,16], long electrical endurance (10^12^ voltage sweeps) [19–24], and long data retention (several years) [25]. RRAM devices can be distinguished on the basis of their switching mechanism into either distributed/local switching or bipolar/unipolar switching [26].

Search for the right functional material for the RRAMs is a significant research area owing to their attractive advantages. The most intensively used RRAM devices to date with high reliability are based on insulating transition metal oxides (TMOs) such as Al_2_O_3_ [10,16,27], TaO_x_ [28], and TiO_2_ [29–32]. A connection is formed between the two electrodes of these TMO-based memory devices in the form of a conductive filament (CF) on the application of external electric field. Switching between the high resistance state (HRS) and low resistance state (LRS) occur due to the formation and dissolution of nanosized filaments. TMO-based RRAMs are further classified into two major categories of valence change memory (VCM) and electrochemical metallization (ECM) based on their physical conduction mechanism [33]. Conduction in VCM is due to the phenomena of oxygen vacancies movement within the TMO active layer while metallic ions from the two electrodes are responsible for causing switching in ECM devices [34]. TaO_x_ based memory devices have in general shown highest stability against the applied voltage sweeps whereas the fastest switching speed and lowest energy losses are the attributes of HfO_x_ based RRAMs [35].

Although TMO-based RRAM devices have exhibited the desired properties, the biggest challenge remains in realizing a functional memory material having all desired attributes in it. For example, the most critical trade-offs are power–speed, speed–retention, and endurance–retention which are not offered by a single TMO-based RRAM device. TMOs based RRAM devices are mostly influenced by surface effects, including surface roughness [36], chemisorption/photodesorption [37], and surface band bending [38]. For example, the electrical properties of TMOs based RRAM devices can be affected by the chemisorption of O_2_ molecules. Its electrical conductivity decreases due to chemisorbed oxygen ions. Furthermore, the existing functional materials being used in RRAMs are facing the bottleneck of scalability. Therefore, search for exploring the new functional materials for RRAM devices remains an open and important research area for realizing the development of commercially available flexible and wearable NVM device of the next generation. 2D materials such as graphene, MoS_2_, hBN, WS_2_, MoTe_2_ and MoSe_2_ [39–56] have already been introduced as the active layer sandwiched between the two conductive electrodes to form RRAM devices on flexible and rigid substrates. Graphene was the first 2D material that was used in electronic device applications followed by its other family members like transition metal dichalcogenides (TMDs), hBN and MXenes. To date, graphene is the leading 2D material due to its electrical and mechanical characteristics required for RRAMs and its ability to be used as a highly conductive electrode material. One of the important characteristics of RRAM devices is their switching ratio ION/IOFF, which can be as high as 10^9^ for 2D-materials-based RRAM devices [57] and is ~10^6^ for TMO-based RRAM [58]. Furthermore, the first TMO-based RRAM device was fabricated around 60 years back in 1967 and compared to them 2D-materials-based RRAM devices have already began to compete with them in only a single decade which is an encouraging sign for the researcher working on these materials. These 2D materials have unique chemical, electrical, thermal, mechanical, physical, magnetic, and optical properties due to their adjustable number of layers that makes a strong case for them to be further explored as the functional layer of RRAMs. The standout feature of 2D materials-based RRAMs is their high flexibility, e.g. the Young’s modulus and yield stress of graphene are 335 N.m^−1^ and 42 ± 4 N.m^−1^ respectively while the bending modulus of MoS_2_ is 9.61 eV [59]. These values of most commonly used 2D materials for RRAM devices suggest that they will offer great flexibility and mechanical endurance. Another important characteristic of 2D-materials-based RRAMs is that they can withstand extreme temperature conditions without compromising on their memory characteristics. 2D-materials-based RRAMs offer highly scalable memory cells with fast switching speed and low power consumption. The switching voltages (SET and RESET) and switching ratio of such devices are also tunable. The detailed discussion about the 2D-materials-based RRAMs has been provided in the later sections of this review paper.

**Figure 1.**
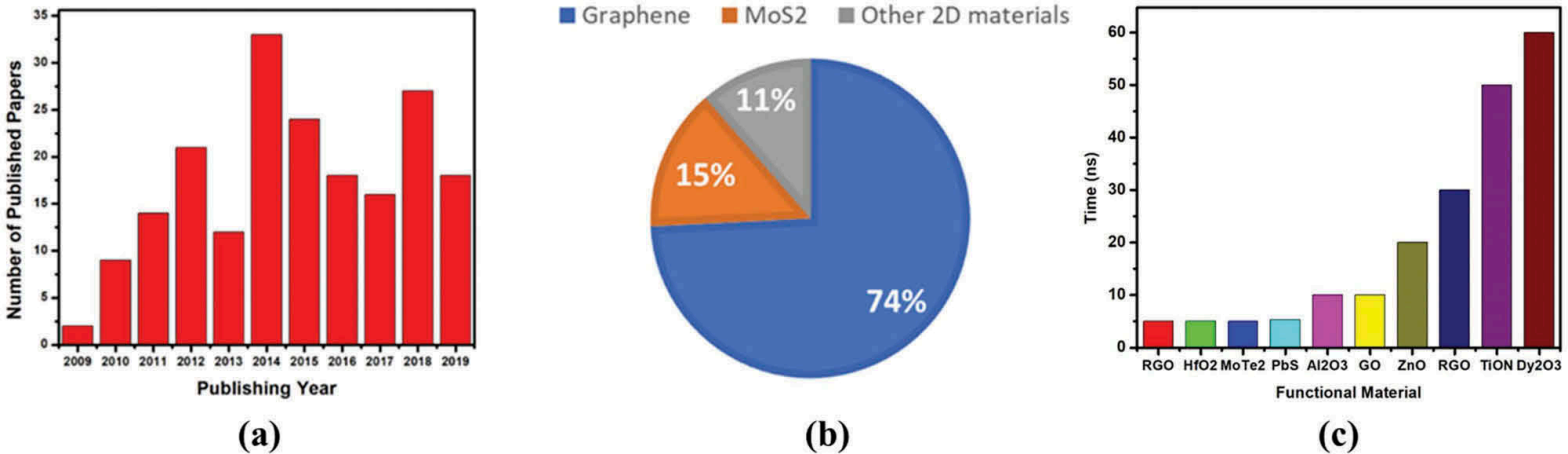
(a) A bar graph illustrating the quantity of published articles on the topic of 2D-materials-based RRAM devices on a yearly basis. (b) A Pi chart showing the ratio of graphene, MoS_2_ and other 2D-materials-based research articles. (c) A bar chart illustrating the high switching speeds of RRAMs based on different functional materials including 2D materials

In this review, we have summarized the resistive switching properties of RRAM devices based on 2D materials and their composites, recent advancements in their performance, fabrication methods, their potential as the future NVM flexible RRAM and challenges faced in realizing its commercialization. Furthermore, research done by each research group has been discussed in detail and their significant contributions have been highlighted to show their importance in the advancement of this topic. The trends of publishing research papers by various research groups in the last decade of 2D-materials-based RRAM devices around the world shows their deep interest and future prospects of these devices as illustrated in Figure 1(a,b) while Figure 1(c) shows the data of a few RRAMs with quickest switching speeds including 2D materials.

## 2. Role of graphene and its derivatives in RRAM devices

### 2.1. Role of graphene in RRAM devices

#### 2.1.1. Graphene as electrode in RRAM devices

Formation and rupture of filaments is one of the most proposal conduction mechanism in RRAM devices. The main problem faced by researchers was to verify this mechanism through advanced technology like transmission electron microscopy (TEM). Graphene-based electrodes resolve this problem owing to theirs high transparency and small thickness, hence allowing the researchers to view the formation and rupture of filaments within the active layer of their devices. Secondly, promising property associated with the RRAM devices is its low power consumption that is often compromised due to the use of traditional electrodes made of metals owing to the contact resistance offered by them but the power losses in graphene electrodes based RRAM devices are low due to its high conductivity and its role as a tunneling barrier. Thirdly, significant contribution offered by graphene electrodes is high scalability due to their ultrathin nature. Moreover, using graphene as an electrode material offers the advantage of mechanical flexibility due to which next-generation nonvolatile RRAM devices can be used in the application of flexible electronics.

**Figure 2.**
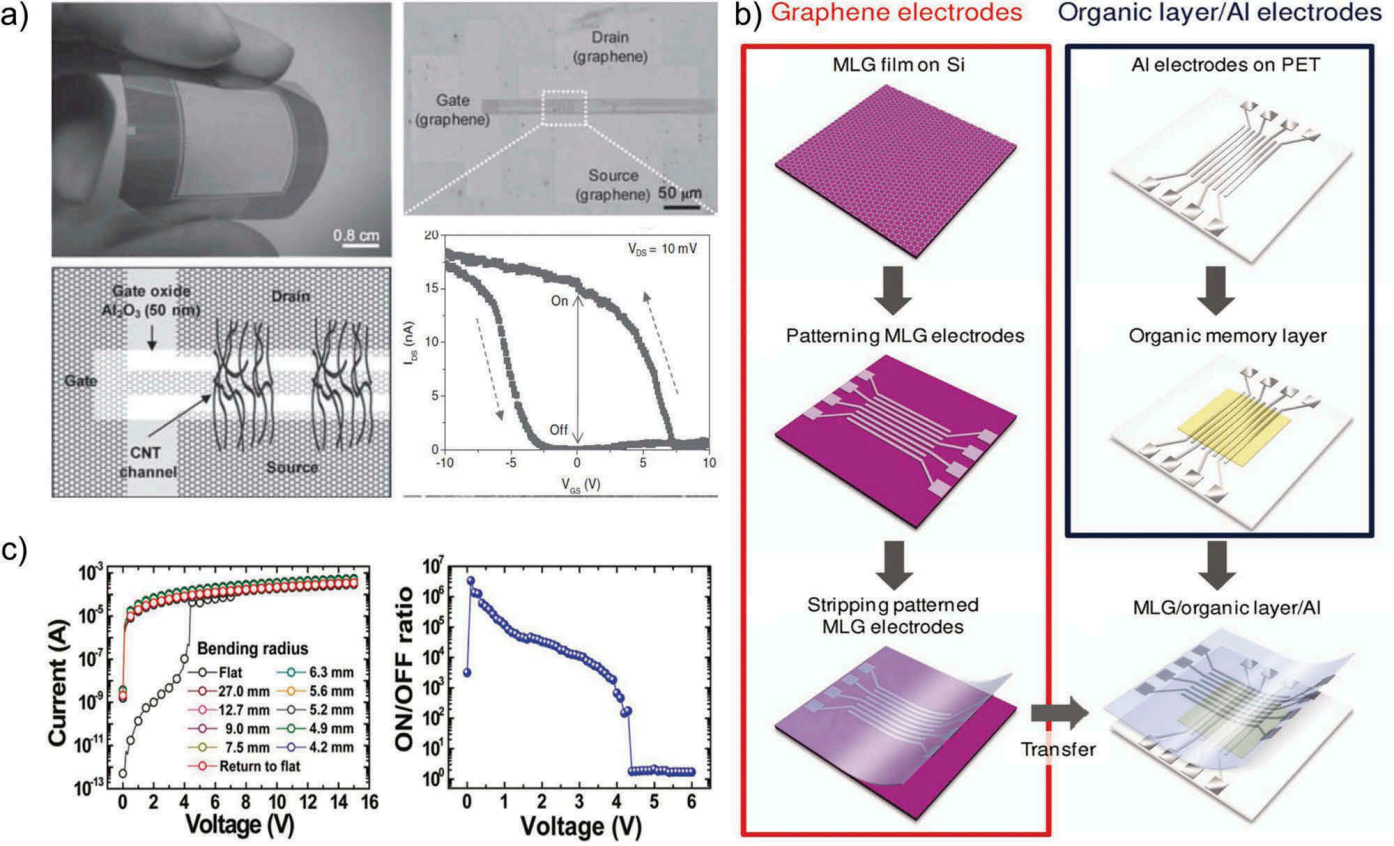
(a) Photograph of the transparent graphene–CNT integrated circuit array (16 × 18) with schematic of the graphene electrode (source, drain and gate) and SWCNT network channel on the PET film. Optical image of a monolayer graphene–CNT transistor (5 channels), in which the dark square rectangle between the source and drain indicates the oxide layer, and Sweeping characteristics of the oxygen-decorated graphene/SWCNT memory device [60]. (b) Fabrication process of flexible organic memory devices with transparent graphene top electrodes. (c) I–V characteristics of an MLG/PI:PCBM/Al flexible organic memory device under different bending conditions and ON/OFF ratio of a flat memory device [61]

Keeping in view all the above-mentioned prospects of graphene, Yu et al. [60] published a significant article in which they explored the resistive switching effect in the flexible and transparent single-walled carbon nanotube (SWCNT) with a distinction that graphene was used as the electrode material. The device was treated with ozone to develop a bonding between oxygen atoms and the surface of graphene that acted as trap sites for charge carriers. The graphene electrodes were decorated with oxygen because it did not decrease the transmittance of electrode as compared to Au and Al nanoparticles that caused a considerable decrease in the transmittance of graphene by 11% and 25%, respectively. This nonvolatile RRAM device based on graphene electrode displayed a high mobility and fast switching speed of ~44 cm^2^ V^−1^ s^−1^, and 100 ns, respectively, with promising mechanical properties (10^3^ bending cycles) owing to the flexibility of graphene electrodes as compared to other rigid metallic electrodes like Ag, Au, Al and Pt. The electrical endurance, retention time and optical transmittance of this RRAM device were 500 voltage sweeps, 10^3^ s and 83.8%, respectively. A photograph of the transparent graphene–carbon nanotube (CNT) integrated circuit array on a polyethylene terephthalate (PET) film, along with an optical image of a monolayer graphene–CNT transistor, is shown in Figure 2(a) while the entire fabrication process of this RRAM device is illustrated in Figure 2(b). Ji et al. [61] reported another important research work on the resistive switching properties of organic memory device fabricated on a flexible PET substrate with graphene electrodes. It was an 8 × 8 bar array with transparent multilayer graphene electrodes. Unlike Yu et al. who used graphene as the bottom electrode for their RRAM device, Ji et al. used multilayer graphene as the top electrode for their organic memory device. This RRAM device exhibited a high switching ratio of 10^6^ and WORM behavior. The mechanical robustness (10^4^ bending cycles) displayed by this device was 10 orders more than Yu et al. with a good cell to cell uniformity and without any prominent decay in device performance. The electrical characteristics of this device are shown in Figure 2(c).

**Figure 3.**
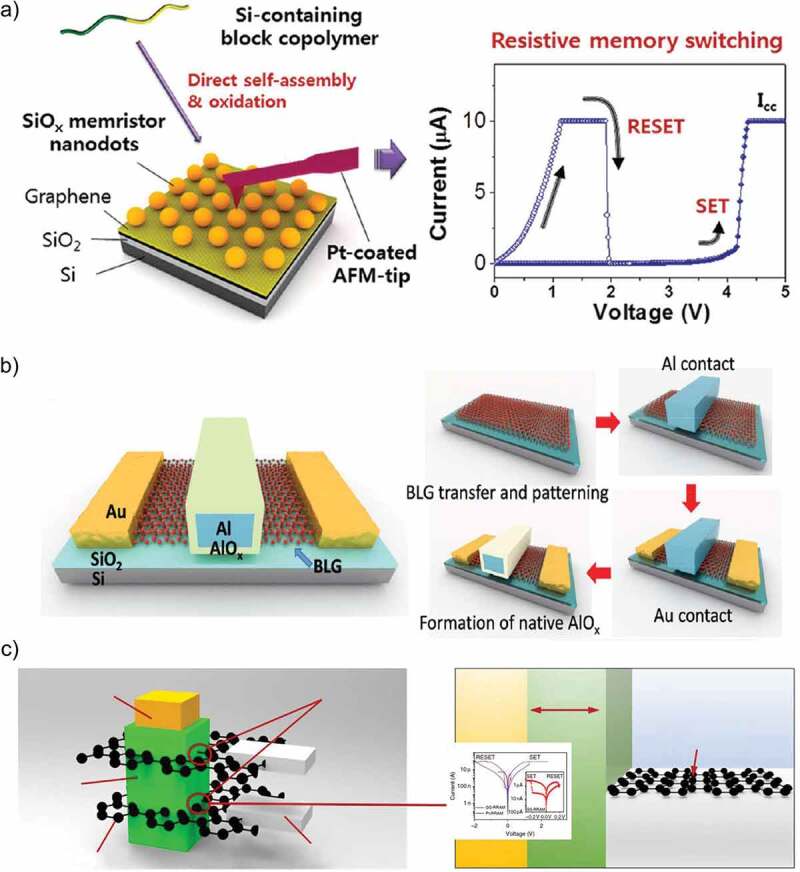
(a) Self-assembled silicon oxide memristors. Lamella-forming and sphere-forming block copolymers were used for preparing thin film and nanodot memristors, respectively. Current (I)−voltage (V) measurement results for the metal/oxide/metal samples and reliability – Pt/SiOx nanodot/Pt [62]. (b) The gate-controlled BLG-based RRAM. The device structure of the GC-GRRAM and the main process flow of the GC-GRRAM [70]. (c) Structure of graphene-based and Pt-based RRAM in a vertical 3D cross-point architecture. An illustration of graphene-based RRAM in a vertical cross-point architecture. The RRAM cells are formed at the intersections of the TiN pillar electrode and the graphene plane electrode. The resistive switching HfO_x_ layer surrounds the TiN pillar electrode and is also in contact with the graphene plane electrode. A schematic cross-section of the graphene-based RRAM with I–V curve [71]

Owing to the increasing interest in RRAM devices and potential of graphene to act as the electrode material in RRAM devices, Park et al. [62] carried forward this work and published an article on the resistive switching behavior of silicon oxide with graphene electrodes. This device displayed unipolar resistive switching behavior that was independent of applied voltage polarity with average memory characteristics of 10^2^ switching ratio and electrical endurance of 80 voltage sweeps. However, the distinguishing factor of this study was the high density of data that could be stored in this RRAM, i.e. up to ~0.5 terabits per square inch. The device was fabricated without using expensive and complex pattern transferring or lithography techniques. A schematic diagram along with the I–V characteristics of the proposed device are illustrated in Figure 3(a). Park et al. did not use the two important features of graphene electrodes, i.e. transparency and flexibility in their proposed RRAM device based on silicon oxide; however, Yao et al. [63] explored both the above-mentioned properties of graphene electrodes in their proposed RRAM device based on silicon oxide on a flexible substrate. They also studied the effect of device size on the conduction mechanism for high-density memory applications. The obtained results of Yao et al. were superior to those reported earlier by Park et al. as the electrical endurance and switching ratio of their memory device were 300 voltage sweeps and 10^5^ respectively. A schematic diagram and the optical image of this device are shown in Figure 3(b).

**Figure 4.**
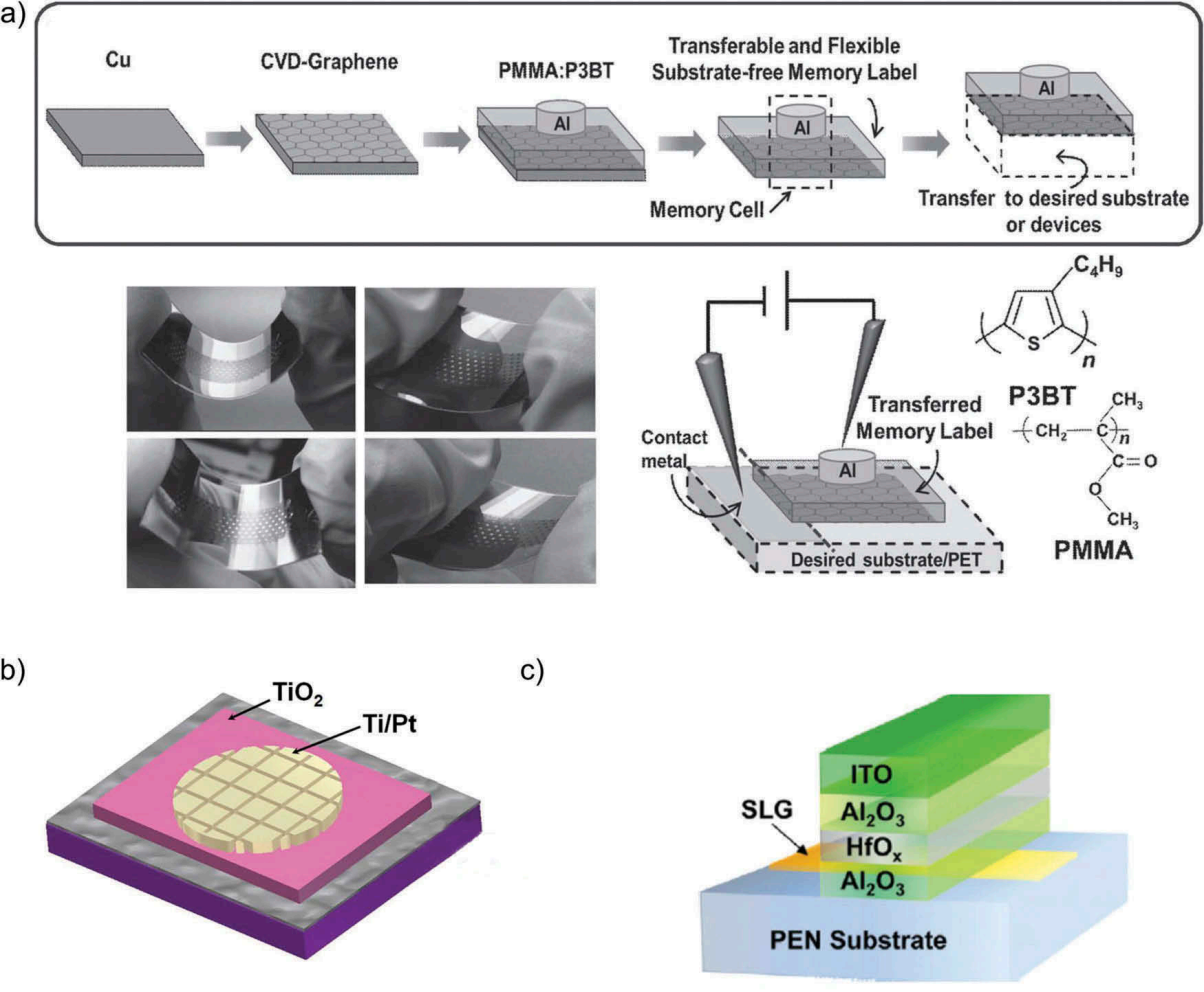
(a) Schematic diagram for the fabrication process of the memory label. Photographs of as-prepared GMML under repetitive bending. Measurement arrangement of the GMML along with the chemical structures of PMMA and P3BT [64]. (b) Schematic drawing of a GMD [65]. (c) Schematic diagram of graphene charge trap memory GCTM device structure [90]

Lai et al. [64] developed a flexible, transferable and sticker type RRAM device with graphene electrodes that could also be developed on arbitrary substrates with high performance. They used PMMA polymer for the dual purposes of insulating active layer of RRAM device along with acting as one of the active layers itself. Poly(3-butylthiophene) (P3BT) was the major active material that was used in this device to store charge. They showed that chemical vapor deposition (CVD)-grown graphene electrodes were very useful for 3D stacking, vertical integration and fabrication on arbitrary surfaces without compromising its high conductivity. They fabricated their RRAM device on various substrates including PET, a vial, medical wristband, glove, silicon wafer, coin and organic diode with one-step transfer process. Their study proves to be an important step forward for the various possible applications such as epidermal electronics and wearable computers. The obtained electrical and mechanical results were impressive, with switching ratio, retention time, and electrical endurance of 10^6^, 10^4^ s and 10^7^ respectively. Photographs of as-prepared GMML under repetitive bending are shown in Figure 4(a).

Owing to the promising attributes of graphene and increasing interest in using it as the electrode for RRAM devices, several groups published their work to explore further insights into this material. Qian et al. [65] fabricated an ultralow power RRAM device on a flexible PET substrate with integrated large area graphene electrodes grown by CVD. The active layer of this memory device was based on TiO_x_. These ultralow power devices did not lose their distinct features of large memory window, high electrical endurance and retention when compared to TiO_x_ RRAM devices with Pt electrodes. This group showed that the heterogeneous interface between TiO_x_ and graphene can both be used for reliable nonvolatile memory purposes with nonlinear electrical characteristics and ultralow power loss (3 orders of reduction as compared to standard metal/oxide/metal structure). Schematic drawing of a GMD device is shown in Figure 4(b).

Different groups contributed in various aspects of RRAM devices by solving different problems related to RRAM devices based on 2D materials. Yang et al. [66] proposed an oxide-based RRAM device with functionalized graphene as electrode in a compact manner. These devices exhibited that functionalized graphene electrodes illustrate an intrinsic threshold switching. The major issue of moderating the sneak paths in RRAM devices to enhance data storage density and scalability was part of this work. Jeon et al. [67] studied the effect of using single-layer graphene (SLG) electrodes on the resistive switching properties of Pt/Al_2_O_3_/TiO_2_/Pt memory device. They solved the problem of observing oxygen ion drift by using transparent graphene as the top electrode. They successfully proved that asymmetric resistive switching in this oxide-based memory device was due to the redox reaction by the oxygen drift at the interface. This device with graphene as the top electrode was electroforming-free with a low threshold voltage of ~1.5 V. Instead of using a SLG electrode, Zhao et al. [68] proposed an oxide-based low-power nonvolatile RRAM device with a multilayer graphene (MLG) electrode. The key features of this device were low threshold voltage (<1 V), low operational current (<100 µA), low power loss (<100 µW), fast switching speed (~60 ns), and high switching ratio (>10^4^). This device also did not require any forming process before carrying out its electrical characterizations. Chakrabarti et al. [69] reported an important device structure of an oxide-based memory device with graphene acting as both the top and bottom electrode to sandwich the tri-layer of TiO_x_/Al_2_O_3_/TiO_2_. This device exhibited forming-free and highly nonlinear switching effect with a high switching ratio of 10^4^. Moreover, the value of its current compliance was extremely low (180 nA) hence, resulting in ultralow power consumption.

Tian et al. [70] exploited the fact that bandgap of a bilayer graphene can be induced through applying vertical electric field. They reported the first ever graphene electrode that was controlled by a gate terminal with a tunable threshold voltage window. This gate terminal was used to induce bandgap in graphene that in turn allowed the electric field to penetrate the active material of RRAM device hence, controlling the quantity of oxygen ions resulting in tuning resistive switching characteristics. Their research group showed that by tuning the operating gate voltage, switching ratio of this graphene-electrode-based RRAM device can be enhanced from 10^3^ to 10^5^. Lee et al. [71] explored the memory effect by exploiting the atomically thin (3 Å) characteristic of graphene by using it as the electrode of a vertical 3D structure RRAM device. They claimed the nonvolatile RRAM device with the lowest power loss and attributed it to the ultrathin graphene electrode. A schematic cross-section of the proposed RRAM and its I–V curve are shown in Figure 3(c). Jang et al. [72] also reported a highly uniform and ultra-low power polymer memory device with MLG like Zhao et al. who used MLG electrodes for oxide-based memory device. This device was based on poly(1,3,5-trimethyl-1,3,5-trivinyl cyclotrisiloxane) (pV3D3) polymer and its cross-sectional HR TEM image is shown in Figure 4(c). Recently, Jang et al. [73] have used as MLG as the bottom electrode for organic-inorganic perovskite on a flexible substrate to resolve the problem of non-flexibility of perovskite materials. Their device displayed asymmetric and reversible bipolar resistive switching behavior in the operating voltage range of 0.68 V to −0.5 V. The standout feature of their device was its mechanical bendability as it was successfully tested for 1000 cycles at a bending radius of 4 mm without any noticeable change in resistive states. The I–V characteristic curves exhibiting its high electrical endurance are illustrated in Figure 10(b) with the inset showing the optical image of the fabricated device. Unlike Jang et al., who used MLG as the bottom electrode, Köymen et al. [74] have reported SLG as the top electrode in the memristive device with a configuration of platinum/TiO_2_/TiO_x_/graphene. The optoelectronic properties of graphene made it a viable choice as a top electrode as it allowed to perform the in situ analysis of the functional layer to a depth of 10 nm. It was due to this reason that they were successful in observing the changes in binding energies at the atomic level to examine the drift of oxygen vacancies. Dugu et al. [75] fabricated two different RRAM devices to study the effect of replacing Pt electrode with a graphene electrode without varying the functional layer and top electrode. They verified that the power losses and threshold voltages of SET-RESET conditions can be reasonably reduced by using a graphene electrode. Ott et al. [76] also used SLG as the top electrode and achieved a switching ratio ten times higher than the metal electrodes with a low power density. This device displayed multi-level resistive states with the possibility of storing more than one bit in a single memory cell. The summary of all the above-discussed RRAM devices with graphene electrode along with their typical characteristics are listed in Table 1.

**Table 1.** Summary of graphene-electrode-based RRAM devices

Bottom electrode	Top electrode	Active layer	Substrate	Switching ratio	Endurance	Retention (s)	References
Graphene	Graphene	SWCNT	PET	10^3^	10^2^	10^3^	[60]
Al	MLG	PI:PCBM	PET	10^6^	10^4^	10^4^	[61]
Graphene	Pt	SiO_x_	Si	10^2^	80	10^4^	[62]
ITO	Graphene	SiO_x_	Glass	10^5^	10^2^	10^5^	[77]
Graphene	Graphene	SiO_x_	Plastic	10^6^	10^2^	–	[77]
Graphene	Al	PMMA:P3BT	PET	10^5^	10^7^	10^4^	[64]
Pt	SLG	Al_2_O_3_/TiO_2_	Si	10^2^	10^3^	–	[67]
Graphene	Ti/Pt	TiO_2_	PEN	10^2^	10^2^	10^6^	[65]
Graphene	Graphene	TiO_x_/Al_2_O_3_/TiO_2_	–	10^4^	10^2^	10^4^	[78]
MLG	MLG	Ta_2_O_5–x_ /TaO_y_	Glass	10^2^	–	–	[66]
Graphene	Graphene	ZnO	Si	10^3^	50	–	[79]
ITO	Graphene	Dy_2_O_3_	Glass	10^6^	10^2^	10^4^	[68]
Graphene	LSMO	BTO	Si/SiO_2_	10^3^	10^3^	–	[80]
Al/MLG	Cu	pV3D3	Si/SiO_2_	20	10^2^	–	[72]
BLG	Al	AlO_x_	SiO_2_	10^3^	–	–	[70]

#### 2.1.2. Graphene as an active layer in RRAM devices

RRAM devices with graphene as their active layer are the potential candidate for high-performance electronics devices owing to their unique mechanical, optical, electrical, and thermal properties because many advanced electronic devices need materials with atomically thin films and wide bandgap. Owing to the compatibility of graphene with the existing planar devices, it can be integrated with CMOS technology. Additional layers of graphene can act as the charge storage medium hence, resulting in higher retention time. Graphene can also offer high transparency, light weight, flexibility and low sheet resistance.

Doh et al. [81] and Shin et al. [82] are considered as the pioneers of using graphene as the active layer of nonvolatile memory device. Both groups fabricated a three terminal device but predicted the possibility of using graphene as the functional layer of a two terminal memory device. The graphene-based RRAM device of Shin et al. exhibited an ambipolar bistable switching behavior with electrical endurance up to 100 voltage sweeps and a small switching ratio of 13. The low value of switching ratio was attributed to the high metal like conductivity of graphene. They also suggested that active layer of graphene can be used for storing multi-bit data and its switching ratio can also be enhanced by inducing a bandgap in graphene layers. Although graphene-based switching device had a drawback of lower switching ratio, owing to its high conductivity, it exhibited fast switching speed. Later, Doh et al. explored the effect of varying active layer thickness on the resistive switching characteristics of graphene-based three terminal devices. The major disadvantage of this device was that its operational voltage was extremely high (>30 V) hence, resulting in a huge power loss. They suggested that the value of operational voltage can be decreased by reducing the film thickness of graphene. Furthermore, the switching ratio of this device was also low, but its retention time was encouraging (2 × 10^3^ s).

After the introduction of graphene as the active layer of a three terminal RRAM device with average properties, Son et al. [83] proposed a flexible bistable memory device which was based on a three-layer structure of organic polymer embedded with graphene. Graphene layers were sandwiched between the two layers of PMMA to provide current stability and enhancing charge storage. This work can be considered as another breakthrough in the graphene-based RRAM devices as it exhibited extraordinary characteristics of a nonvolatile memory device. The switching ratio, electrical endurance and retention time for this device were 1 × 10^7^, 1.5 × 10^5^ cycles and 1 × 10^5^ s, respectively. Based on the trend of obtained results, Son et al. predicted that their device could have the lifetime of 10 years. The operating voltage (± 2 V) of this device was also much lower as compared to the three terminal device based on graphene proposed by Doh et al. thus, resulting in less power losses. The conduction mechanism of this device was attributed to the classic theory based on the formation of conductive filaments. Other than the above mentioned fascinating traits of this hybrid device, it was the first memory device based on graphene active layer that was fabricated on a flexible substrate. These devices were bent up to a bending radius of 10 mm without any prominent decay in their performance. This group suggested that embedding a complete layer of graphene in between the organic polymers instead of nanoparticles has added advantages of reproducibility and higher flexibility due to uniform distribution of graphene.

**Figure 5.**
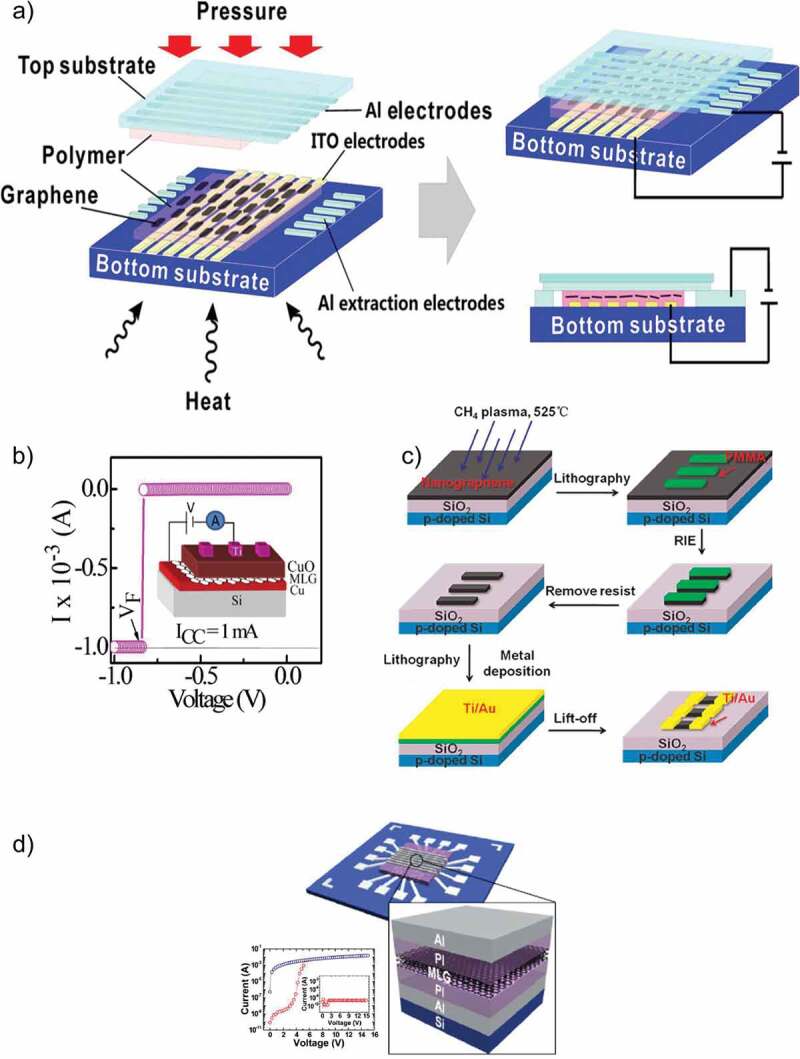
(a) Schematic diagram of the hybrid bistable memory devices fabricated utilizing graphene sheets sandwiched between polymer layers [85]. (b) Current-voltage characteristics of Ti-CuO-MLGCu sample during initial electroforming step showing transition to low resistance state (LRS) at forming voltage (VF) of 0.84 V with a current compliance limit (ICC) of 1 mA. Inset shows a schematic view of Ti-CuOMLG-Cu structure [86]. (c) Schematic diagram of the fabrication process for the two-terminal devices. Uniform and large-area graphene film on SiO_2_ (300 nm)/Si substrates was used as starting materials; PMMA resist mask was patterned by e-beam lithography; removal of the exposed graphene by reactive ion etching; removal of the PMMA overlay in hot acetone; contact electrodes formation by a second-step e-beam lithography and Ti/Au (2 nm/30 nm) deposition by e-beam evaporation; lifting-off of the metals for the two-terminal resistive switching devices [89]. (d) Geometry of the organic ferroelectric-graphene memory device, where W is the width and L is the length of the graphene channel [96]

More research work was carried out on the graphene-based resistive switching device by Akdim et al. [84] who proposed the switching behavior of graphene nanoribbons. Although the switching characteristics were not that impressive as compared to the work of Son et al., their distinctive contribution was to explore the effect of uniaxial strain on graphene-based RRAM device. Wu et al. [85] proposed another hybrid memory device similar in structure to the one proposed earlier by Son et al. with a layer of graphene sheets embedded in between two layers of polymers. The main difference in their structure was that instead of using two similar polymers, they used two different polymers of polystyrene (PS) and polyvinyl-carbazole (PVK). This device was write-once-read-many-times memory (WORM) with a volatile behavior. The operating voltage of this device was relatively high, in the range of ± 6 V, as compared to its predecessors; however, the achieved switching ratio of this hybrid device was like the one reported by Song et al. (1 × 10^7^). The fabricated device exhibited an average reproducibility of 64% as only 16 out of 25 cells operated appropriately. Schematic diagram of this hybrid bistable memory device is shown in Figure 5(a).

Later that year, Singh et al. [86] proposed a bipolar memory device based on the hybrid interface of copper oxide and MLG. Graphene acted as the blocking layer and a storage medium for oxygen anions. This device required electroforming at an applied voltage of −0.84 V before its proper functioning. A high switching ratio of 1 × 10^3^ with a low operating voltage of ± 0.8 V was recorded for this hybrid device. No prominent decay in its electrical performance was observed for 100 voltage sweeps. It was deduced that the bilayer structure of Ti-CuO-MLG-Cu exhibited bipolar resistive switching behavior while I–V curves of Ti-CuO-Cu displayed only a rectifying behavior. Their results clearly showed that resistive switching in this hybrid structure was due to the inclusion of MLG without which their devices displayed rectifying behavior instead of memory behavior. The major drawback of this device was that it required a preliminary electroforming step that was attributed to migration of oxygen anions from CuO and induced electric field generation. Another important result studied by this group was the impact of ambient and vacuum conditions on the resistive switching characteristics. It was deduced that the values of reset voltage and current were significantly reduced to 0.07 V and 1 mA as the operating conditions switch from ambient to vacuum environment due to the availability of more oxygen anions in vacuum conditions. I–V characteristics of this device during initial electroforming process displayed a forming voltage (VF) of 0.84 V with current compliance current value of 1 mA as shown in Figure 5(b) with its schematic diagram shown in the inset.

More articles related to the role of graphene as the active layer in resistive switching devices were published in the year 2012 owing to the promising characteristics of this 2D material as reported in the previous years. Wu et al. [87] reported commendable results with a switching ratio of a single graphene sheet reaching up to 10^6^. They listed an important drawback of using the hybrid active layer approach earlier reported by Son et al. that due to the inclusion of organic polymers, degradation time of these devices can be quick due to the vulnerability of organic materials towards environmental conditions like humidity. The main difference in this device from the previous graphene-based memories was its structure as it was a two terminal device with a planar structure instead of the typical sandwich structure. Due to a larger planar gap between anode and cathode (4 µm) as compared to the sandwiched devices, its operating voltage was on a higher side (±7 V) with a drawback of higher power losses. Like their previously reported sandwiched structure hybrid memory device based on PS/graphene/PVK, this planar structured device also displayed WORM behavior. A second substrate at the top of the active layer of graphene sheet was used to keep it in contact with the planar electrodes during high current state. After the reports of Son et al. and Wu et al. on the fabrication of memory devices based on hybrid active layer, Ji et al. [88] developed another nonvolatile memory device based on the hybrid structure in which layers of graphene were sandwiched between two layers of organic polyimide (PI) polymers with a configuration of PI/MLG/PI. The reason for repeating this approach of embedding graphene layer in between two polymer layers was to produce high-quality results. The switching ratio achieved by this group was a little lower compared to some earlier reports, but it was still superior to memory devices based pure graphene (>10^6^) with a decent retention time (10^4^ s). Although all these reported results with hybrid active layer approach achieved results that were close to each other, the major difference of this device as compared to the previously reported devices was the selection of a different set of organic polymers and electrodes. The memory device by Ji et al. exhibited excellent cell to cell distribution that was verified by their cumulative probability plots. The fabrication of this device was relatively more difficult and complex as it employed a greater number of steps and was time-consuming. They also proved that memory behavior in this hybrid device was due to the charge trapping ability of MLG sandwiched layer. A schematic diagram of the fabricated memory device is shown in Figure 5(d).

He et al. [89] proposed another remarkable graphene-based memory device with extremely promising characteristics of a nonvolatile RRAM. Their study was comprehensive and instead of using the typical sandwiched structure, He et al. also choose the planar structure for their device just like the two terminal memory devices of Wu et al. A lot of new insights were created in this work such as instead of only two resistive states, this RRAM device had multilevel resistive switching with a total of five different states. The switching speed and yield of this device were the other two prominent features with the values of 500 ns and 98%, respectively. Moreover, the gap between anode and cathode was in nanoscale instead of microscale hence resulting in extremely low operating voltage and reduction of power losses that was a drawback of the microscale planar memory device proposed earlier by Wu et al. The drawback of this RRAM device was that it required an electroforming step (V_break_ = 5.5 V) before applying a double voltage sweep across its electrodes to cause electrical breakdown. Unlike the typical RRAM memory devices, this device neither showed unipolar or bipolar memory behavior; instead, it exhibited nonpolar resistive switching behavior independent of voltage polarity. A schematic diagram of the fabrication process for the two-terminal device is shown in Figure 5(c).

Kim et al. [90] recognized the supreme flexibility present in the monolayer of graphene and fabricated a memory device on a transparent polyethylene naphtalate (PEN) substrate. Only 8% loss in transparency of PEN substrate was observed due to the presence of highly transparent graphene layer on its surface. Their device had a memory window of ~8.6 V with 30% retention/10 years. Their device displayed highly stable and repeatable bistable resistive states under successive compressive and tensile stress cycles. The proposed RRAM device by Kim et al. based on graphene had a potential to be used as a flexible and transparent electronic memory device with high endurance under flex.

Lai et al. [91] proposed a memory device based on graphene nanoflakes (GNF) assisted macromolecular composite memory device with a low operating voltage. They proposed a solution-processable functional material that was a blend of GNF and poly(vinyl alcohol) (PVA). This work was the first report on the direct integration of graphene into any RRAM device through a low-temperature and single-step processing technique. Operating voltage of this device was relatively low (−1.3 V to +1.4 V) with a remarkable electrical endurance of 10^7^ voltage cycles. The major advantage of the graphene-based RRAM device proposed by this group was to achieve excellent switching properties through an extremely simple and cost-effective solution-process method with an active area of 3 mm^2^.

Another important work was reported by Shindome et al. [92] as they thoroughly investigated the conduction mechanism taking place in graphene-based RRAM devices by varying the number of graphene layers, changing device size and varying the contact metals. Monolayer, six-layer and eight-layer graphene was used as the functional layer in the form of a planar structure. They used standard microscopy techniques of atomic force microscope (AFM), electron energy-loss spectroscopy (EELS) and cross-sectional TEM to examine the conduction mechanism of their memory device and concluded that it is based on the chemical bonding-state change of graphene nanoribbons. Yang et al. utilized the highly transparent property of graphene to fabricate a fully transparent RRAM device based on ZNO active layer. They proved that combining graphene with ZNO had a significant impact on the resistive switching properties with not only enhanced transparency but also reversible resistive switching by suppressing the surface effects. Graphene absorbed only ~2% of incident light.

**Figure 6.**
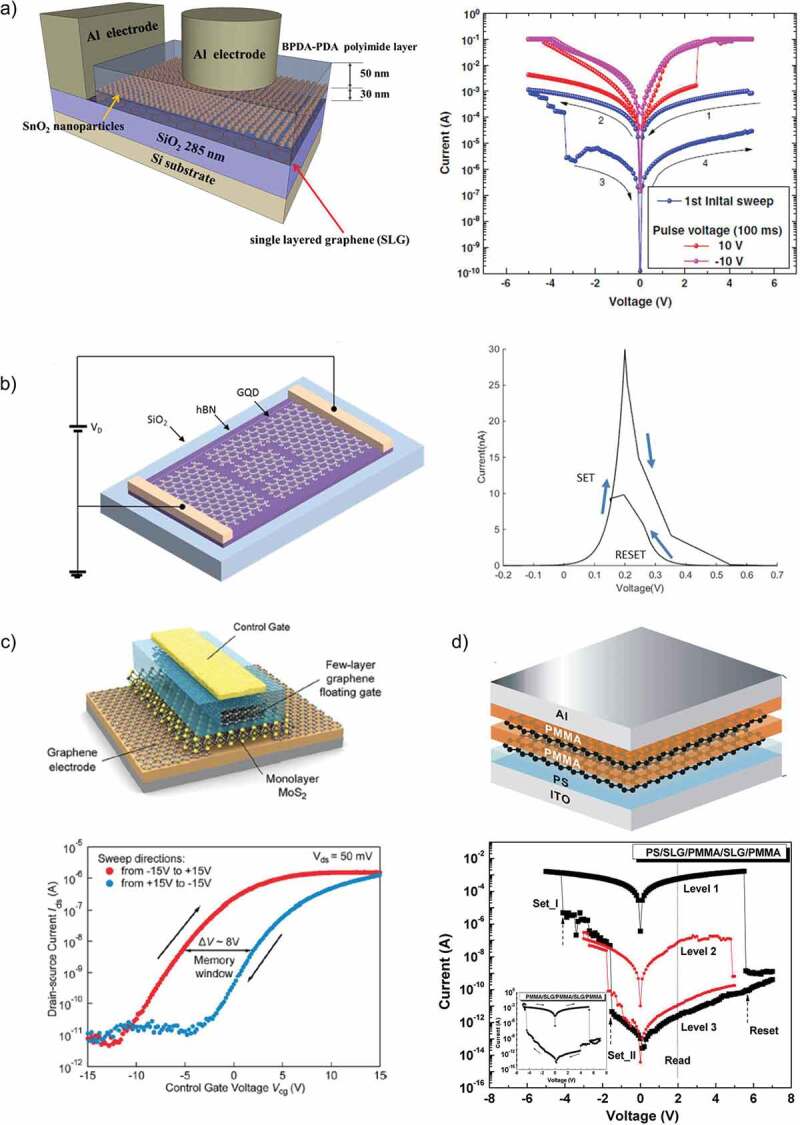
(a) Schematic 3-dimensional structure of memory device with SnO_2_ nanoparticles embedded in BPDA-PDA polyimide layer on SLG contact layer. I–V curves of the resistive switching memory with SnO_2_ nanoparticles embedded in BPDA-PDA polyimide layer on SLG contact layer [94]. (b) Circuit and schematic diagram of the GQD RRAM device along with the simulated I–V characteristics of the device [113]. (c) Electric hysteresis loop of graphene ferroelectric memory. R as a function of top gate voltage for the graphene-ferroelectric sample and Schematic of graphene floating gate FLASH memory [97]. (d) The magnifying schematic structure of the ITO/PS/SLG/PMMA/SLG/PMMA/Al memory unit cell. I–V curves of ITO/PS/SLG/PMMA/SLG/PMMA/Al device and I–V curve of ITO/PMMA/SLG/PMMA/SLG/PMMA/Al device [101]

Berzina et al. [93] reported another RRAM device based on the thin film of polyaniline-graphene composite by applying the Langmuir-Schaefer method. They analyzed the variations in electrical properties such as S-like I–V curves and increased operating voltage range that were associated with accumulation of charges in graphene sheets. A schematic representation of the organic memristor with its I–V characteristics are shown in Figure 6(b). The trend of embedding metal oxide nanoparticles in a polymer layer on a SLG was pioneered by Lee et al. [94] when they reported an interesting active layer of SnO_2_ embedded BPDA-PDA polyimide on SLG. However, this experiment was not successful as it displayed a maximum switching ratio of only 7.9 with a relatively higher operating voltage of ± 5 V. A schematic diagram and I–V curve of the proposed RRAM device are shown in Figure 6(a). A significant result was reported by He et al. [95] as they explored the tunable electroluminescence (EL) effect in the planar structure RRAM device based on graphene/SiO_2_. They concluded that this relation between the EL and resistive states can be used to distinguish between both. Their device could be used for the possible applications of optical communications.

Wang et al. [97] published a research news on the progress of graphene-based nonvolatile memory devices in which they acknowledged the substantial impact of graphene on resistive switching memory devices but emphasized on further development in this field to reach a sizeable impact. They suggested that in graphene-based memory devices one should be careful about response time and power consumption of their memory devices. Furthermore, they pursued that low cost and highly reliable fabrication methods are the need of hour for achieving high throughput memory devices. Furthermore, it was suggested by Wang et al. that a lot of research efforts were required to explore the nanoscale memory devices to realize their integration with CMOS systems. They rightly raised the hope of a flexible memory device based on the promising results achieved by graphene RRAM cells. Electric hysteresis loop of graphene ferroelectric memory and its schematic diagram are shown in Figure 6(c). Rocci et al. [98] reported a nano hybrid based memory device based on a few layer graphene deposited on the planar structure of manganite electrodes grown on SrTiO_3_ substrate. These devices exhibited hysteric transport and rectifying resistive switching behavior as depicted from their I–V curves with the ability of working properly in the temperature range of 10 K to 300 K. The memory effect in the single crystalline multilayer and monolayer-graphene-based nano switches was explored by Li et al. [99] These devices had the ability to be operated at a pull-in voltage of 1 V with a high switching speed of ~100 ns and an electrical endurance of 5000 voltage sweeps that was better than other reported graphene-based nanoelectromechanical resistive switches. Monolayer graphene-based device exhibited lower pull-in voltage while the bilayer-graphene-based device showed higher speed.

Impressive switching results in a tri-layer structure of graphene flakes sandwiched between two layers of polyvinylidene fluoride (PVDF) polymer were reported by Khurana et al. [100]. This tri-layer geometry was further sandwiched between indium tin oxide (ITO) bottom electrode and Pt top electrode to exhibit multilevel resistive switching for storing high data density. They found that Fowler-Nordheim and space charge limited current mechanisms were responsible for resistive switching effect in these devices. These graphene-based tri-layer memory devices showed highly stable resistive switching effect in four different states with a high retention time (10^4^ s) and good electrical endurance (150 biasing cycles). Although such a device structure with a sandwiched graphene layer between two polymers layer had already been used by Son et al., Wu et al., and Ji et al., but the major differences of the proposed RRAM device by Khurana et al. is the difference in selected polymer material causing a multilevel resistive switching with a distinguished advantage of high-density data storage.

Wu et al. [101] proposed a multilevel resistive switching behavior in a different device structure based on single-layer graphene. The proposed device configuration consisted of five layers with the configuration of polymer/SLG/polymer/SLG/polymer sandwiched between two metal electrodes. Although the switching ratio (10^4^) of this device was more than the one reported by Khurana et al., it had a drawback of complex structure and use of excessive materials as it contained two extra layers. A schematic diagram and obtained I–V curve of the fabricated device with multi-resistive states are illustrated in Figure 6(d). Furthermore, it consisted one less resistive state hence resulting in lower data storage. Atab et al. [102] illustrated that the memory behavior can be enhanced in ZnO-based RRAM device by embedding the graphene nanoplatelets in it. Graphene nanoplatelets acted as the charge trapping material that also helped in lowering the threshold voltage hence resulting in low power consumption due to its high conductivity value. Inclusion of graphene nanoplatelets also enhanced the retention time up to 10 years with 25% data loss. Later that year, Cristea et al. [103] reported the impact of embedding graphene quantum dots (GQDs) in the organic polymer of poly(3-hexylthiophene) (P3HT) on its resistive switching characteristics. They also verified that GQDs act as the charge trapping material in the polymer-based memory device. Inclusion of GQDs greatly enhances the hole transport. The P3HT-GQDs-based RRAM device had a planar structure with a long conduction channel of 8 µm and two Au electrodes acting as anode and cathode.

**Figure 7.**
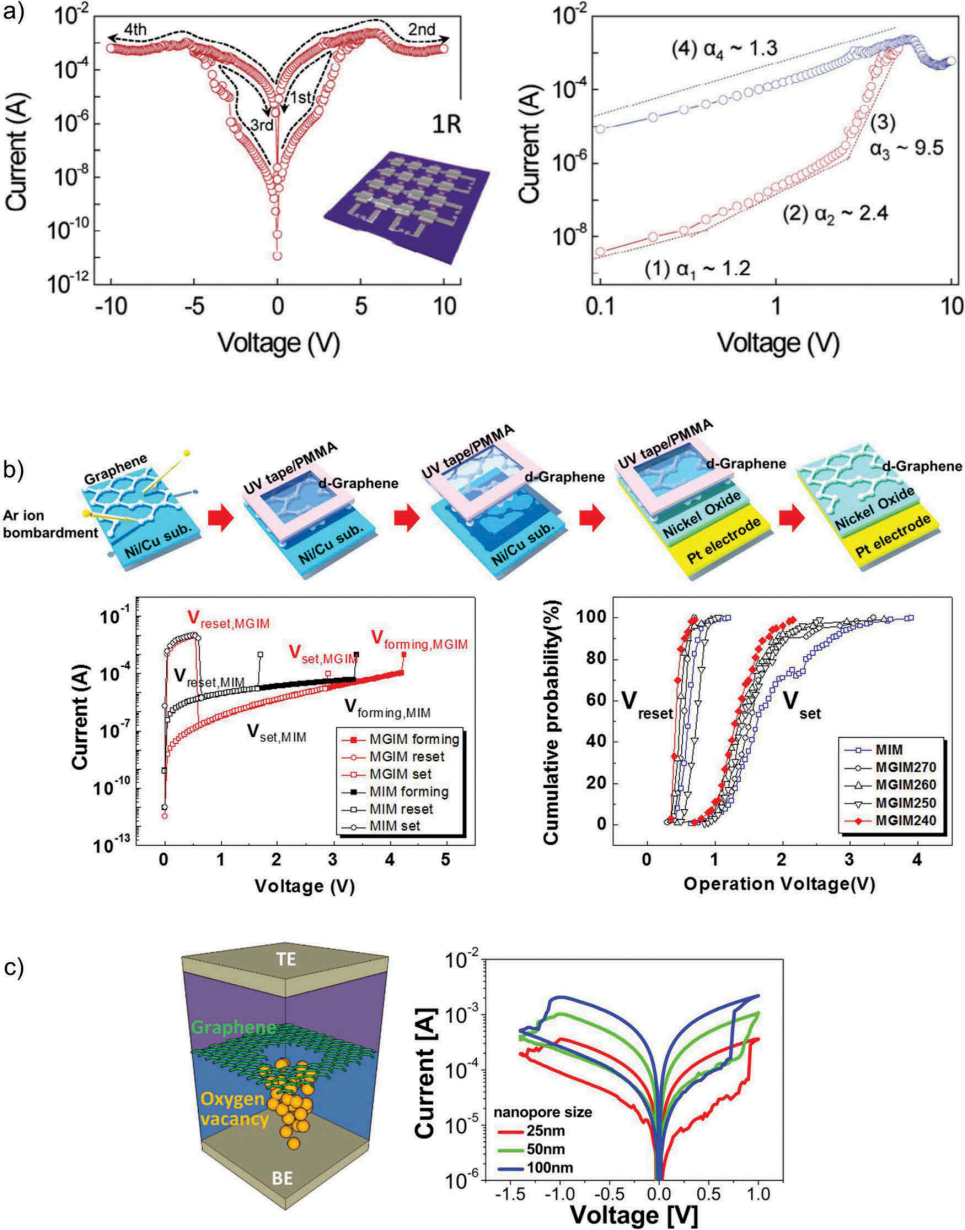
(a) I–V characteristic of memory-only (1 R) device and its Log–log plot of I–V characteristics at 300 K [107]. (b) Resistive switching characteristics of MGIM structures compared with a conventional MIM structure. Illustration of fabrication process for MGIM structure. D-graphene is made before it is transferred to device. Initial current-voltage characteristics of the MGIM and conventional MIM structures. Cumulative probability of switching voltages, V_set_ and V_reset_, for MGIM structures with d-graphenes irradiated with Ar+ ions at kinetic energies of 240 eV (MGIM240), 250 eV (MGIM250), 260 eV (MGIM260) and 270 eV (MGIM270) as well as a MIM structure [105]. (c) Schematic of the graphene-inserted memristor structure where oxygen ions transport only through a nanopore created in the graphene layer, forming a CF with oxygen vacancies with I–V curve [109]

Our research group explored the resistive switching effect in the nanocomposite of poly(4-vinylyphenol) (PVP) and graphene nanocomposite thin films. The obtained results were significant in a way that different approaches of device structure were studied including blended and non-blended functional thin film. It was concluded that blended functional thin film of PVP-graphene exhibited better stability and higher switching ratio as compared to using these two materials as a bilayer [104]. As a continuation of exploring the resistive switching effect of this nanocomposite, it was fabricated on a flexible PET substrate to evaluate its mechanical robustness. The devices were bendable up to a bending diameter of 1.8 cm for 50 bending cycles without any prominent decay in its performance. Lee et al. [105] illustrated that the resistive switching characteristics of an oxide layer can be enhanced by simply inserting a defective graphene layer. The impact of this graphene layer was that it reduced the random rupture of filaments hence resulting in stability of the obtained results. Furthermore, the value of threshold voltages was also reduced that resulted in lower power losses. The whole fabrication process and the obtained electrical characteristics of the proposed RRAM device are illustrated in Figure 7(b). The memory effect in the polymer-suspended graphene nanoplatelets was reported by Kang et al. [106]. Although the device exhibited reliable performance, its switching ratio was only 10.

Ji et al. [107] investigated the resistive switching behavior of organic polymer after mixing it with the nanocomposite of ZnO-GQDs and exhibited their use as an active element in combination with a diode-one resistor. They obtained average memory results with a high reliability by using ZnO-GQDs nanocomposite acting as charge trap sites. The architecture used in this work can be very effective to reduce or eliminate the cross talk between consecutive memory cells. They tested their devices through encoding a complete word by using the standard ASCII code. A schematic diagram, its I–V curve and double logarithmic I–V curve are illustrated in Figure 7(a).

Kurkina et al. [108] explored the switching effect in the partially fluorinated graphene thin films. Lee et al. [109] found the ionic transport mechanism in memory devices of graphene sheets with engineered nanopores. They tuned the resistive switching characteristics of tantalum oxide material by inserting a graphene film with engineered nanopores. The role of graphene was to block redox reactions and ionic transport. The effect of different sized nanopores was also studied and it was depicted that value of LRS and HRS were directly proportional to the nanopore size of graphene. The effects of annealing on the ion migration due to engineered nanopores of graphene were also studied at a high temperature of 300°C. Schematic of the graphene-inserted RRAM structure in the graphene layer, along with its I–V curve is illustrated in Figure 7(c). Mannequin et al. [110] reported the resistive switching effect in the interface of graphene and HfO_2_. They compared the device performance of HfO_2_ device with and without graphene layer between the top electrode and found out that an immense improvement in the retention of ON state occurred by adding graphene layer due to its oxygen reservoir effect.

A very different and significant study was reported by Ueda et al. [111] in which they reported the photo-controlled memory effect in the heterojunction of graphene/diamond with multilevel resistive states. The obtained results showed that only blue and violet light had the ability to switch the resistive states with a maximum switching ratio of 10^3^ even at a high temperature of 200°C. These results clearly illustrated that the heterostructure of graphene/diamond have the potential to be used as either a photo switch or for photometry purposes. Among those regular research papers, a review article was written by Rani et al. [112] to discuss the conduction mechanism taking place in the nonvolatile RRAM devices based on graphene due to the fact that researchers were taking keen interest in using it as the active material for RRAM devices. This article contains ample amount of information for those readers who are interested to use graphene as either an active layer or electrode in the RRAM device.

Pan et al. [113] fabricated a two-terminal and a three-terminal RRAM device based on GQDs with resonant tunneling and volatile memory behavior. Addition of the third terminal was useful to modify the resistive switching states. They also used branch and load resistors with this device to obtain different hysteresis characteristics. Introduction of third gate terminal induced more logic states for each memory cell. Kapitanova et al. [114] have explored the photomemristive effect in the heterojunction of self-assembled nanoscale graphene oxide/graphene. These devices were photosensitive with ultra-high capacity nonvolatile memory and multilevel resistive switching. These devices were functional both in dark conditions and under the effect of light. Recently, Shehata et al. [115] used a nanocomposite of graphene and TiO_2_ in a planar device as the functional layer of RRAM device. Nanoparticles of TiO_2_ were uniformly dispersed on the surface of graphene sheets by using UV irradiation that helped in enhancing the trap sites for the electrons. The I–V characteristic curve and its schematic diagram of the planar structure are shown in Figure 10(a).

Liu et al. [116] fabricated a graphene-based RRAM device with extremely low power consumption of less than a femtojoule by using the weak van der Waals forces of its surface and suggested their device for the possible application of neuromorphic computing systems. Sun et al. [117] successfully eradicated the negative set response of ITO/methacrylate epoxy resin (MER)/Al-based RRAM device through the addition of graphene blocking layer. This result was achieved due to the resistant nature of graphene layer that does not allow the conductive filament to diffuse into the inert ITO electrode. Wu et al. [118] realized that by the addition of graphene layer in a binary transition metal-oxide-based memory device, its volatile behavior can be changed to nonvolatile with high stability and the ability to store multiple bits in a single cell. Yalagala et al. [44] developed a memory device based on the composite of MgO-PVP-Graphene and used it for the security-based applications. The distinctive feature of this study was that the whole device was water dissolvable in less than 3 s owing to the high hydro-dissolution rates of PVP and MgO. They proposed a novel feature of destroying the memory from their device wirelessly from a remote place. The summary of all the above discussed graphene-based RRAM devices along with their typical characteristics are listed in Table 2.

**Table 2.** Summary of RRAM devices with graphene as the active layer

Bottom electrode	Top electrode	Active layer	Substrate	Switching ratio	Endurance	Retention (s)	References
Ti/Au	Au	Few-layer graphene (FLG)	Si/SiO_2_	–	–	10^3^	[81]
Cr/Au	Al	Graphene	SiO_2_	–	10^2^	–	[82]
ITO	Al	PMMA/graphene/PMMA	PET	10^6^	10^5^	10 years	[83]
Al	ITO	PS/graphene/PS	Glass	10^7^	3	10^6^	[85]
Al	ITO	PVK/graphene/PVK	Glass	10^4^	5	10^2^	[85]
Cu	Ti	MLG/CuO	Si	10^3^	10^2^	–	[86]
Ti/Au	Ti/Au	Graphene	SiO_2_	10^3^	10^4^	10^5^	[89]
Al	Al	PI/MLG/PI	Si	10^6^	–	10^4^	[88]
ITO	ITO	Graphene	Glass	10^6^	–	10^4^	[87]
Ti/Cr/Au	Ti/Cr/Au	Graphene nanoribbons	Si/SiO_2_	10^6^	10^2^	10^3^	[92]
ITO	Ag	GNF:PVA	Glass	10^2^	10^7^	10^4^	[91]
SiO_2_	Al	Graphene/BPDA:PDA	Si	7.9	–	10^4^	[94]
Ti/Au	Ti/Au	Graphene	Si/SiO_2_	10^5^	–	–	[95]
ITO	Pt	PVDF/Graphene/PVDF	Glass	10^5^	50	10^4^	[100]
Au	Au	P3HT:GQDs	Si	–	–	–	[103]
ITO	Al	PS/SLG/PMMA/SLG/PMMA	Glass	10^4^	–	10^4^	[101]
ITO	Ag	PVP-Graphene	Glass	5	20	–	[104]
Al	Al	ZnO-GQDs	SiO_2_/Si	10^3^	10^2^	10^4^	[119]
ITO	Ag	Graphene/PVP	PET	35	40	10^2^	[120]
NiO	Pt	Graphene/NiO	Ni/Cu	10^2^	10^2^	10^5^	[105]
Pt/Ti	Al	ZnO-GQDs	SiO_2_	10^3^	10^3^	10^4^	[45]
ITO	Au	Al_2_O_3_/Graphene	Glass	10^3^	10^2^	10^4^	[75]
ITO	Al	Graphene/MER	PET	10^3^	40	10^4^	[117]
Pt	Ag	Graphene/HfO_x_	–	10^7^	10^3^	10^3^	[118]
Ag	ITO	MgO-PVP-Graphene	PET	10^3^	10^2^	10^4^	[44]

### 2.2. Role of reduced graphene oxide/graphene oxide in RRAM devices

**Figure 8.**
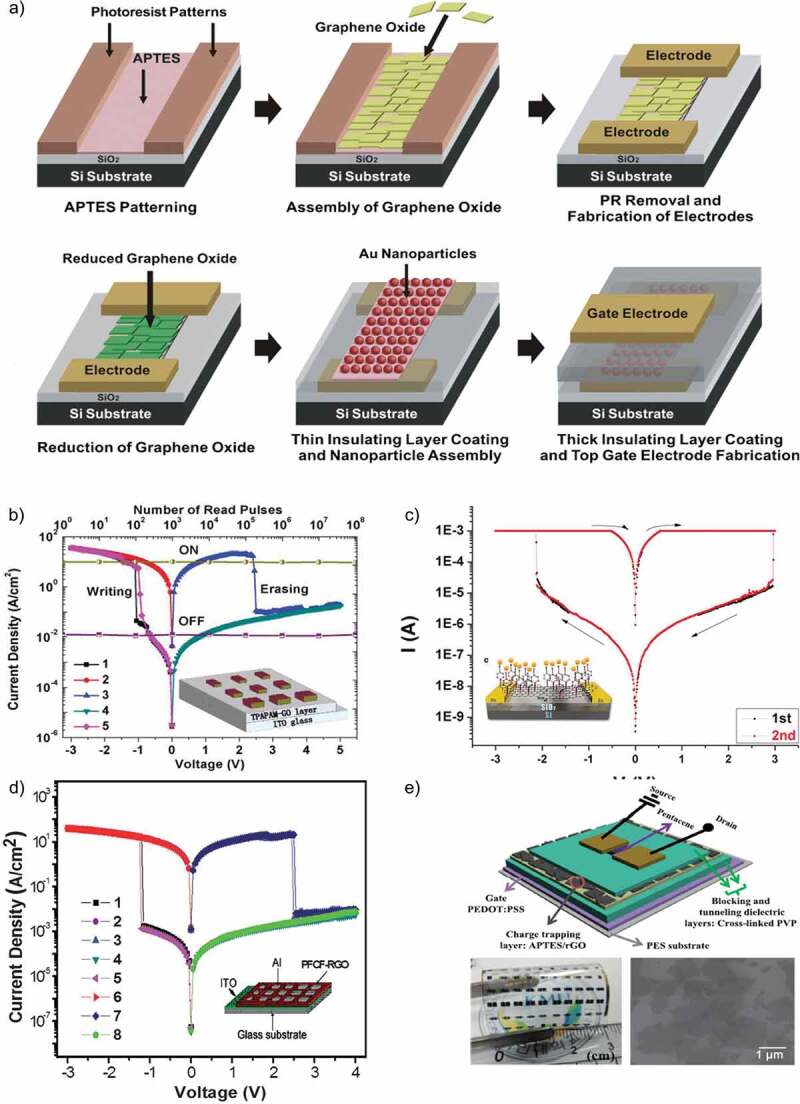
(a) Schematic diagram depicting the basic fabrication process of reduced GO-based memory devices. Patterning APTES SAM on the SiO_2_ substrate using photoresist patterns. Assembly of GO pieces onto APTES layer in solution. Removal of photoresist and fabrication of electrode. Reduction of graphene oxide. Deposition of Al_2_O_3_ via ALD, patterning APTES and OTS SAM layers on the Al_2_O_3_ layer using microfabrication, and the assembly of Au NPs onto the APTES SAM regions. Here, OTS was used for passivation. Deposition of a thick Al_2_O_3_ layer followed by the fabrication of top-gate electrodes [128]. (b) J–V characteristics and stability tested in either ON or OFF state under stimulus by read pulses of a 0.16-mm^2^ ITO/TPAPAM-GO/Al device. Inset: schematic diagram of the single-layer memory devices [129]. (c) I_V characteristics on log scales of the vertical device (i.e. Al top-electrode/AuNP-fRGOs hybrid material film/ITO bottom-electrode). Noticeable nonlinear hysteresis is observed in all devices [122]. (d) Current density/voltage (J/V) characteristics of a 0.16 mm^2^ Al/PFCF-RGO/ITO device; the symbols indicate sweeps 1–8 [130]. (e) Three-dimensional schematic illustration of RGO-based organic transistor memory device. Photograph of the fabricated flexible RGO transistor memory devices. SEM image of RGO on the APTES-coated substrate [131]

Nonvolatile and bistable memory devices based on Graphene Oxide (GO) have prompted great interest due to its high optical transparency, high flexibility, low cost, easy fabrication, environmentally friendly nature, controllable physical and chemical properties, for future transparent and flexible electronic devices. Vasu et al. [121] in 2011 reported the unipolar resistive switching effect in the ultra-thin films of reduced graphene oxide (RGO) on a rigid glass substrate with a high yield of >99%. The obtained switching ratio of 10^5^ with extremely fast switching speed of 10 µs was much higher than most of the graphene-based (GO) memory devices. This RGO-based RRAM device was compared with a MWCNT-based RRAM device whose switching ratio was much lower (400) with huge power losses as its threshold voltage was 20 V. Later, Cui et al. [122] studied the nonvolatile memory behavior of the covalently bonded gold nanoparticles embedded in RGO thin film. They also observed the effect of device structure on its properties by fabricating both sandwiched and planar structure device with the same active layer. The devices displayed stable results of multiple write-read-erase cycles (10^3^ cycles) with high retention time (700 s) while the switching ratio exhibited by the sandwiched structure device was higher than the planar structure. A schematic diagram and I–V characteristic curve of the proposed device is shown in Figure 8(c).

Hu et al. [123] in 2012 published a research article in which they controlled electron transfer effect in RGO that was noncovalent functionalized with thionine. The obtained results were promising with a fast switching of <5 ns, switching ratio of 10^4^, and retention time of 10^5^ s. This study provided a new method for regulating the electronic characteristics of graphene for RRAM devices. In the same year, Liu et al. [124] published an important article in which they explored the nonvolatile memory behavior and high flexibility of RGO RRAM device. This was the first ever RRAM based on RGO that was fully solution-processed and flexible. In this RRAM device apart from the active layer, both electrodes were also made of RGO making the device a low cost and environment-friendly RRAM. Like earlier reported RRAM devices based on RGO, this RRAM device also had an undesired feature of high threshold voltage (±14 V). The flexibility of the RRAM device was tested for 1000 bending cycles without any prominent decay in its performance. Seo et al. reported the rewritable and nonvolatile memory behavior in the partially reduced graphene oxide doped with nitrogen. They mainly targeted the drawback of RGO-based memory devices, i.e. uncontrollable oxygen functional groups. In order to induce stability in the obtained results, the functional layer was doped with nitrogen. They deduced that the memory behavior of partially reduced GO was highly dependent on the concentration of doped nitrogen and the RRAM device completely lost its memory behavior with decreased concentration of nitrogen atom.

**Figure 9.**
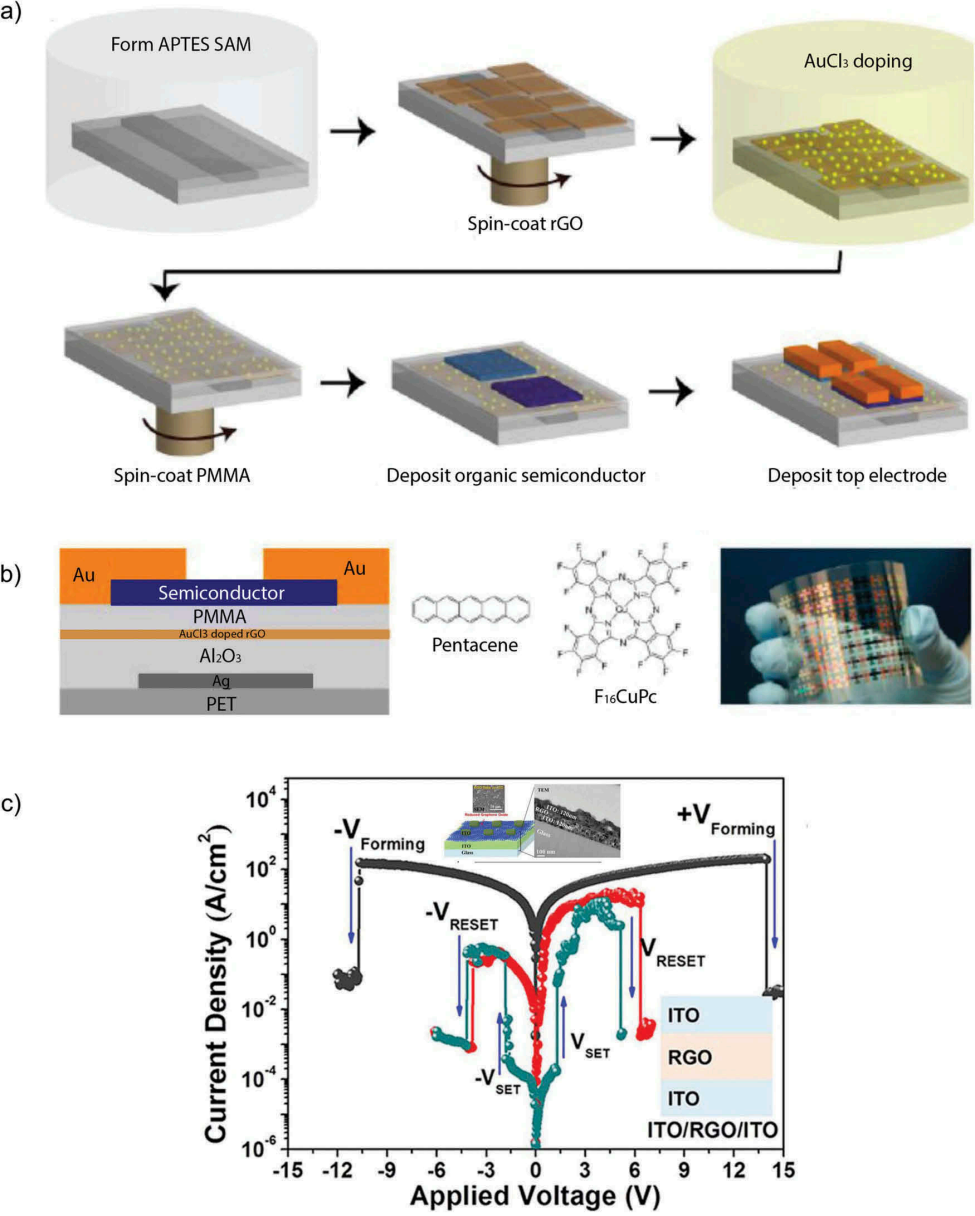
(a) Schematic diagram depicting the basic fabrication process of AuCl_3_ doped RGO floating gate memory device. (b) Fabricated device structure for memory device with inserting AuCl_3_ doped RGO floating gate and molecular structure of pentacene and F_16_CuPc. The optical image of the flexible memory [125]. (c) I–V characteristics of the ITO/RGO/ITO device; the inset shows the ITO/RGO/ITO structures [127]

Han et al. [125] investigated the tunable memory characteristics by engineering the energy band through controlled doping of RGO. As the functional layer was doped with gold chloride (AuCl_3_), a gradient increase in the value of RGO work function was noticed. They demonstrated a flexible RRAM device with a large memory window and controllable threshold voltages. The basic fabrication process is shown in Figure 9(a), along with a schematic diagram of device structure, and its optical image is shown in Figure 9(b). Ho et al. [126] studied the switching mechanism of RGO active layer through impedance spectroscopy. They compared the results of RGO with pristine GO active layer to figure out the possible conduction mechanism responsible for resistive switching behavior. They deduced that memory behavior in RGO is due to the oxidation and reduction reactions taking place at the top interface between oxygen migration in the active layer and Al electrode. The retention time for this device was >10^6^ s that was the highest among all other RGO-based memory devices reported till then and the operating voltage was also low (±4 V). However, despite better operating voltage and retention time, its switching ratio was inferior to the previously reported RRAM devices. Later that year, Kim et al. [127] published an article and reported the multilevel resistive switching behavior in reduced graphene oxide sandwiched between two ITO electrodes by fabricating a transparent memory cell. This RRAM device exhibited an optical transmittance exceeding 80% with 00, 01, 10, and 11 resistive states. Multilevel resistive states were achieved by varying the amplitude of applied pulse voltage from 2 V to 7 V. The device displayed high endurance and retention time of 10^5^ s each at a temperature of 85°C. The I–V characteristics of this multiresistive RRAM device with its schematic diagram are shown in Figure 9(c). Myung et al. [128] proposed an ambipolar RRAM device with a planar structure and RGO embedded with Au nanoparticles as its active layer. The addition of Au nanoparticles caused a huge increase in the size of hysteresis window. This device exhibited an n-type and p-type behavior in the negative and positive applied bias region, respectively. The fabrication procedure of this device is illustrated in Figure 8(a). Zhuang et al. [129] reported the nonvolatile and bistable memory effect in the GO layer functionalized by a conjugated polymer of triphenylamine-based polyazomethine (TPAPAM) with the device configuration of ITO/TPAPAM-GO/Al. The obtained I–V characteristics were commendable with an electrical endurance of 10^8^ and a switching ratio of 10^3^ as illustrated in Figure 8(b). Zhang et al. [130] proposed another RGO-based RRAM device grafted with conjugated polymer poly- [{4,4’-(9H-fluorene-9,9-diyl)bis(N,N-diphenylbenzenamine)}{4-(9H-carbazol-9-yl)benzaldehyde}(9,9-dihexyl-9H-fluorene)] (PFCF-CHO) with a device configuration of ITO/PFCF-RGO/Al. The switching ratio (10^4^) and electrical endurance (10^8^) of this device were the standout features of this device. The I–V curves of first 8 voltage cycles along with a schematic diagram are shown in Figure 8(d). Rani et al. [131] proposed a RGO-based memory device with a transistor-like device structure on a flexible substrate. Their low cost and facile fabrication process-based memory device exhibited nonvolatile memory behavior with an electrical endurance, and retention time of 10^2^ and 10^5^ s, respectively. The device structure in the form of its schematic diagram along with its optical image is illustrated in Figure 8(e).

Lin et al. [132] investigated the resistive switching behavior of ZnO RRAM device with a capping layer of RGO layer. They depicted from the obtained results that RGO insertion brought stability to the memory behavior of ZnO with a better switching ratio of 10^5^. They stated that the layer of RGO played the role of oxygen reservoir in this RRAM device hence, making the transportation of oxygen ions easy. Another important advantage of inserting RGO layer was that it restricted the oxygen vacancies to react with Al electrode. This device displayed the highest electrical endurance of 10^8^ voltage sweeps as compared to all other RRAM devices based on reduced graphene oxide. In the same year, Midya et al. reported the resistive switching effect in a different composite of reduced graphene oxide blended with PVA polymer embedded with Au nanoparticles. Although they obtained average results, their work opened the gates for other researchers to explore the memory behavior in other hybrid nanocomposites of RGO.

Finally, Pradhan et al. [133] published an important article in which they resolved the problem of high power losses in RGO-based RRAM devices by fabricating a low power nonvolatile RRAM device with a threshold voltage even less than 1 V. Earlier the lowest operating voltage for RGO-based RRAM device was 4 V achieved by Ho et al. They also examined the effect of device size, film thickness and scan time on the switching properties of the as fabricated memory device. The resistance value had a direct relation with temperature owing to which they deduced that the conduction mechanism of this device was due to the formation and rupture of metallic filament.

Cao et al. [134] mixed the 2D material of RGO with a novel and highly soluble polymer-based on organophosphorus to form a device configuration of Al/PTPEB-g-RGO/ITO. Although Midya et al. also reported the resistive switching effect in a hybrid nanocomposite of RGO with a polymer earlier on, Cao et al. achieved superior results with a switching ratio exceeding 10^4^ and a retention time of 10^4^ s.

Chen et al. [42] reported a dual nature memory device based on the bilayer structure of GO and polyoxometalates (POMs) with the features of bipolar resistive switching and WORM. The transition between both memory behaviors was attributed to the change in the barrier at the interface of GO and POM. Their work was an important contribution as it discussed a lot of insights about the tunable memory characteristics and design of GO-based hybrid memory devices. Zhang et al. [135] also proposed a hybrid memory device based on GO and a polymer with the configuration of Al/PVK-AZO-GO/ITO. The main contribution of this team was also based on suggesting the new design of a GO-based nonvolatile memory device with the feature of multiple storage. Another hybrid composite based on the mixture of a polymer (PI) and GO layer was proposed by Choi et al. [136] by using a simple fabrication technique that illustrated a WORM behavior. The switching ratio achieved by this group was extraordinary as it exceeded 10^8^ which was ascribed to the high charge storage capacity of GO.

Wan et al. [137] also proposed a hybrid memory device of GO layer but instead of using a polymer as the second material, they preferred to use a perovskite oxide of strontium titanate (SrTiO_3_). SrTiO_3_ nanoparticles were embedded in the functional layer of RGO and the obtained results illustrated an interesting mechanism in which the resistive switching behavior of the fabricated device changed from digital to analog. The digital memory effect was due to the formation and rupturing of filaments inside the functional layer of SrTiO_3_ on the application of applied voltage; however, the memory effect was tuned to analog after the addition of RGO layer as Ag filaments were not easily broken on the application of negative voltage. Kim et al. [138] proposed another important study for understanding and improving the conduction mechanism of in GO-based memory devices due to their potential of future nonvolatile memory device. They used an in situ TEM technique to observe the chemical and physical changes in the bonds of nanoscale GO sheets and oxygen owing to the application of biased voltage. They clearly observed that a conical-shaped dynamic conductive graphitic channel grows from the upper region with more oxygen region to the lower region with less oxygen. Qi et al. [139] also worked on the conduction mechanism of GO-based memory device and engineered a new way to change the volatile memory nature of GO-based memory device to a stable bipolar resistive switching by embedding oxidized carbon quantum dots (OCQDs). They also attributed the switching mechanism in their device to the migration of oxygen vacancies.

They verified a definite increase in the retention of low resistive state due to the addition of OCQDs after studying the effect of temperature on GO-OCQDs nanocomposite-based functional layer. They enhanced retention was due to the increase in barrier height that restricted the migration of oxygen. Such devices can be very useful in reducing the power consumption of future nonvolatile memory. Srivastava et al. [140] used the finite element modeling (FEM) to observe the effect of GO and RGO as a bilayer in TiO_2_ based memory device. Their study showed that GO acted as the channel for the formation of filaments; however, RGO acted as an electrode instead of a functional layer. This work contained experimental results backed by the theoretical results obtained by the simulation. Saini et al. [141] studied the effect of annealing on the bipolar resistive switching behavior of GO layer at different values of temperature ranging from 100°C to 400°C. They also used the TEM technology and observed the formation of aluminum filament from top to bottom electrode. The maximum switching ratio of 10^4^ was observed at an annealing temperature of 200°C.

Shi et al. [142] observed the complementary resistive switching in GO-based memory device. They studied the effect of varying compliance current on the quantity of oxygen defects for the formation of conductive channels within the functional oxide layer. They reported that complementary resistive switching behavior was only observed when the magnitude of rest voltage was greater than that of set voltage. Songkeaw et al. [143] fabricated a completely transparent WORM device by forming a composite of poly (ethylene-co-vinylacetate) with GO. The optical transmittance of the formed memory device in the visible light was 60% with a high switching ratio of 10^5^ and good retention time under variable temperature conditions.

**Figure 10.**
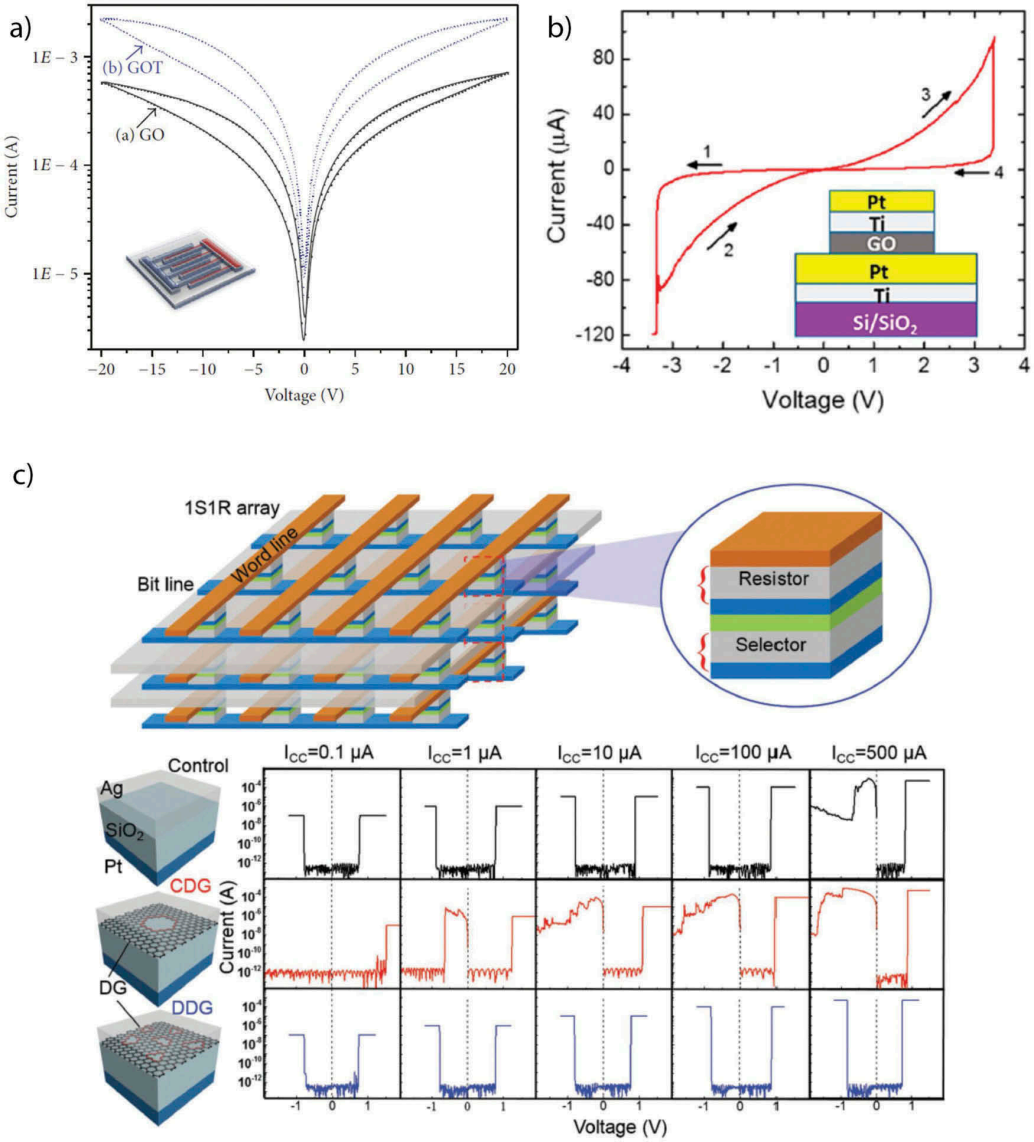
(a) Current-voltage characteristics of graphene oxide (GO) and graphene oxide/TiO_2_ (GOT) devices; the inset is the planar structure of prepared devices [115]. (b) Resistive switching characteristics after repeated bending tests up to 1000 cycles at R = 4 mm [73]. (c) 3D 1S1 R scheme, cell structures and typical I–V characteristics of the control, CDG, DDG devices. Schematic illustrations of the 3D 1S1 R crossbar array where the RS memory (resistor) and the accessory selector are indispensable components in an operational cell. Cell structure and typical RS I–V characteristic comparison of the control (first line), CDG (second line) and DDG device (third line) under various ICC. The maximum volatile ON-state currents are 100, 0.1 and 500 [145]

Recently, Kim et al. [55] fabricated a multi-stacked RRAM device based on the sandwiched composite layer of GO-PVA between the two insulating polymeric layers of PVA. They also observed the effect of changing temperature like Saini et al. on their device by changing its value from 100°C to 200°C. Interestingly the switching ratio observed by this group at 200°C was also 10^4^ as reported by Saini et al. earlier. This work seemed to be not contributing much in the advancement of achieved results based on GO RRAMs with a drawback that it used several layers hence, increasing the cost and difficulty level of device fabrication. In the ongoing year, Li [144] has also shared his tunable results of solution-processable GO-based memory device mixed with the polymeric layer of polymethyl methacrylate (PMMA) by using the theory of charge trapping. The tunable characteristics of this device were controlled by varying the concentration of GO (0.5–5 wt%) in the insulating layer of PMMA. They observed that 0.5 wt% of GO did not have any impact on the OFF state characteristics of PMMA-based RRAM device; however, the overall conductivity of the active layer began to increase. Interestingly the device with the higher content of GO (2 wt%) showed a lower switching ratio that can be attributed to the fact that by increasing the content of GO, value of high resistance/OFF state kept on decreasing and hence, resulting in lesser gap between the two resistive states. The device with the maximum content of GO, i.e. 5 wt% showed completely conductive behavior without any memory effect. Zhao et al. [145] solved the problem of current retention in carbon-cation-based memory devices that is associated with the deteriorating stability of conductive filaments. The deterioration occurs by decreasing the compliance current value hence causing the high amount of power losses. This group solved this problem by controlling distribution of centralizing and decentralizing the conductive filaments. The managed to control the size and quantity of these filaments by inducing a defective graphene layer in this structure as shown in Figure 10(c). The summary of all the above-discussed GO/RGO-based RRAM devices along with their typical characteristics are listed in Table 3.

**Table 3.** Summary of GO/RGO-based RRAM devices

Bottom electrode	Top electrode	Active layer	Substrate	Switching ratio	Endurance	Retention (s)	References
Pt	Cu	GO	Ti/SiO_2_/Si	20	10^2^	10^4^	[101]
ITO	Al	GO-PVK	Glass	10^3^	10^8^	10^4^	[146]
ITO	Al	GO	Glass	10^3^	10^2^	10^9^	[147]
ITO	Al	TPAPAM-GO	Glass	10^3^	–	10^4^	[129]
Al	Al	GO	PET	10^2^	10^2^	10^5^	[148]
ITO	Al	AuNP-GO	Glass	10^2^	–	10^2^	[122]
ITO	Al	RGO	Glass	10^5^	–	–	[121]
ITO	Al	GO	Glass	10^3^	10^2^	–	[149]
Pt	Pt	GO/PCMO	SiO_2_/Si	10^2^	–	10^4^	[150]
ITO	Ag	GO-PI	Glass	10^5^	10^2^	10^3^	[151]
ITO	Al	PFCF-RGO	Glass	10^4^	10^8^	10^4^	[152]
ITO	Al	BCP-GO	Glass	10^5^	10^8^	10^4^	[153]
Pt	Pt	GO	SiO_2_/Si	10^4^	10^2^	10^5^	[123]
GO	GO	GO	PET	10^2^	10^3^	10^3^	[124]
Pt	Al	GO	Si	10^4^	10^2^	10^3^	[154]
ITO	Ag	GO	PET	5	–	10^3^	[155]
Ag	Ag	GO	SiO_2_	10	–	10^3^	[156]
PEDOT:PSS	Au	PVP/APTES/RGO/PVP/pentacene	PES	–	10^3^	10^7^	[131]
ITO	Al	GO	PET	10^2^	10^2^	10^4^	[149]
ITO	Au	GO	–	10^2^	–	10^3^	[157]
ITO	Al	RGO	SiO_2_	15	–	10^3^	[158]
ITO	Al	GO-PANI	Glass	10^3^	10^8^	10^4^	[159]
ITO	Al	TiO_2_/GO	Glass	10^2^	10^5^	10^3^	[160]
ITO	ITO	RGO	Glass	10^3^	10^5^	10^7^	[127]
Ag	Au	RGO	PET	10^4^	10^2^	10^5^	[125]
Al	Al	RGO	Glass	10	10^2^	10^6^	[126]
ITO	Ag	GO	–	10^5^	–	10^3^	[161]
ITO	Al	GO-CISQDs	Glass	10^4^	10^5^	–	[162]
ITO	Al	GO-ZnO	PET	10^2^	10^2^	10^4^	[163]
Ta	Pt	GO/Ta_2_O_5-x_	Si/SiO_2_	10^2^	10^2^	–	[164]
Al	Al	ZnO/RGO	SiO_2_	10	10^3^	10^5^	[132]
ITO	Al	RGO-PVA-AuNPs	PET	10^3^	50	10^3^	[165]
ITO	Al	GO-AuNPs	Glass	10^8^	–	10^4^	[166]
ITO	Au	GO	Glass	10	10^2^	–	[167]
Al	Al	PS/GO/PS	Si	–	10^6^	–	[168]
ITO	Al	PVDF-GO	Glass	–	–	–	[169]
ITO	ITO	GO	PES	10	–	10^5^	[170]
ITO	Al	GO-AuNPs	Glass	10^5^	10^2^	10^4^	[171]
Al	Al	GO	SiO_2_	–	–	–	[172]
Al	Al	GO	Glass	10^2^	10^2^	10^4^	[133]
ITO	Al	PVK:GONPs	PET	10^2^	–	–	[173]
Ti/Pt	Ti/Pt	GO	Si/SiO_2_	10^3^	10^4^	10^5^	[174]
ITO	Al	PMMA/GO	Glass	10^3^	10^4^	10^5^	[175]
ITO	Al	PTPEB-g-RGO	Glass	10^4^	10^2^	10^4^	[134]
ITO	Al	PW/GO	Glass	10^3^	10^2^	10^4^	[42]
ITO	Al	PVK-AZO-GO	Glass	10^4^	10^6^	10^4^	[135]
FTO	Ag	GO/SrTiO_3_	Glass	15	10^4^	10^4^	[137]
Pt	Pt	RGO	Si/SiO_2_	10^5^	–	–	[138]
Au	Au	PVA/GO-PVA/PVA	Glass	10^4^	10^2^	10^3^	[55]
ITO	Ni	PMMA:GO	Glass	10^3^	10^2^	10^6^	[144]
ITO	Al	PI-GO	Glass	10^8^	10^2^	10^3^	[136]
ITO	Al	OCQDs-GO	–	10^2^	10^2^	10^5^	[139]
ITO	Al/Au	GO	Glass	10^4^	–	–	[141]
ITO	Al	GO	Glass	–	–	–	[142]
ITO	ITO	EVA/RGO	PEN	10^5^	–	10^4^	[143]

## 3. Role of MoS_2_ in RRAM devices

The potential of NVM effect in MoS_2_ was first explored by Liu et al. [176] when they illustrated the resistive switching effect in a hybrid nanocomposite of MoS_2_-PVP sandwiched between RGO and aluminum. It was observed that the bipolar resistive switching shown by this device was due to the trapping and detrapping of charge carriers from MoS_2_ flakes blended in organic polymer (PVP). Although MoS_2_-PVP memory device exhibited average performance with a switching ratio of 10^2^ and operating voltage of ±5 V, it illustrated the potential of another 2D material to be used as the active material for flexible memory device of the future.

**Figure 11.**
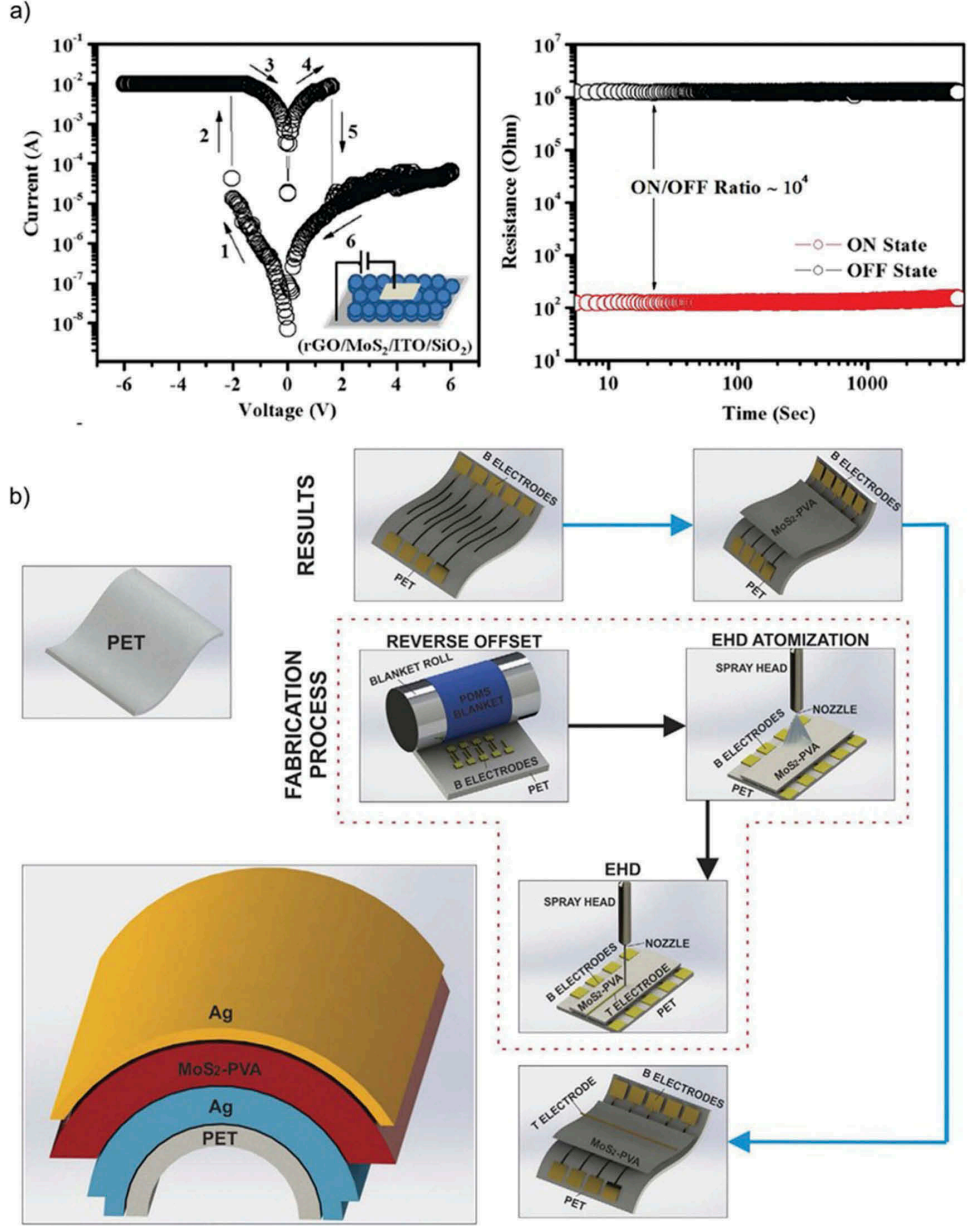
(a) I–V characteristic curve and schematic diagram (inset) of the RGO/MoS_2_/ITO memory device along with its retention graph [177]. (b) Schematic diagram illustrating the whole manufacturing process of as fabricated memory device on a flexible PET substrate. The images enclosed in the red dotted block exhibit each step of the fabrication process while the images outside the block correspond to the resulting device after each step. The bottom left corner exhibit layer by layer sandwiched structure of a single memory cell in bend state with PET/Ag/MoS_2_- PVA/Ag configuration [8]

After the successful experiments by Liu et al., Xu et al. [177] reported another important study in which instead of MoS_2_ flakes blended with another material, they used only MoS_2_ in the form of nanospheres as the active layer sandwiched between RGO and ITO electrodes on a rigid substrate of Si. Unlike the average switching properties shown by the RRAM device based on MoS_2_ flakes, MoS_2_ nanospheres exhibited excellent memory behavior with a higher switching ratio (10^4^), lower operating voltage (±2 V) and a retention time of 10^4^ s. These achieved results were attributed to the unusual electrical properties of nanosphere assemblies. Later, Sun et al. [178] explored the bipolar resistive switching effect in the single layer of MoS_2_ sandwiched between fluorine-doped tin oxide (FTO) and Ag. Although the switching ratio of this device was lower as compared to the one reported by Liu et al., the device exhibited an extremely low operating voltage (±0.4 V) with electrical endurance up to 100 voltage sweeps that is highly desirable as it reduces the overall power consumption. Bessonov et al. [179] proposed a solution-processed bilayer structure of MoS_2_ and molybdenum oxide (MoO_x_) displaying a characteristic bipolar resistive switching behavior with multilevel resistive states and very high switching ratio of 10^6^. This was an extremely important study in the context that the operating voltage (±0.2 V) and switching ratio (10^6^) of this MoS_2_-based memory device were far superior to several other devices based on either graphene or MoS_2_. Moreover, the fabrication technology was completely based on printing techniques that made the device fabrication extremely simple, quick and cost-effective. A schematic diagram of this device along with its electrical characteristic curves are illustrated in Figure 11(a).

Sangwan et al. [180] reported the bipolar resistive switching effect in the monolayer of MoS_2_ with tunable characteristics of gate-controlled device in a field effect geometry. The memory phenomena in this device were mediated by the grain boundaries in the monolayer of MoS_2_. The distinctive factor of these devices was that it displayed negative differential resistance (NDR) behavior. The threshold voltage of these devices was tunable from 3.5 V to 8 V with a variation in the applied voltage at the gate terminal. This distinctive structural change provided a new opportunity for designing bipolar and complementary switching devices.

Cheng et al. [181] explored a unique aspect of MoS_2_ sheets by studying the effect of varying MoS_2_ phase on its electrical properties. They proved that the 2 H phase of bulk MoS_2_ had ohmic behavior while the 1 T phase of exfoliated MoS_2_ sheets exhibited a characteristic bipolar resistive switching effect when sandwiched between two Ag electrodes. This device showed the least operating voltage value of ±0.2 V that was earlier achieved by Bessonov et al. as well. A similar study by Zhang et al. [182] was reported shortly after the work of Cheng et al. in which they also studied the effect of 1 T and 2 H phase of MoS_2_ with a difference that instead of using MoS_2_ alone, they used a hybrid nanocomposite of MoS_2_-PVP that was earlier reported by Liu et al. This work can be thought as a combination of the study done by Liu et al. and Cheng et al. with an important additional feature that these devices were fabricated on a flexible PET substrate. Zhang et al. concluded that 1 T-MoS_2_ and 2 H-MoS_2_ devices exhibited two different types of NVM behaviors including WORM and rewritable memory, respectively. Wang et al. [183] proposed a photoresistive bistable memory device based on MoS_2_ nanospheres. The control of this device was based on the polarization of nanospheres in light and dark conditions. Although the switching ratio of that device was only ~10, multilevel resistive switching effect was achieved through varying illumination intensity of white light. No resistive switching effect was observed at zero light intensity/dark conditions. Our research group also reported the characteristic bipolar resistive switching behavior in the novel nanocomposite of MoS_2_ flakes interspersed in the organic polymer of PVA to form a hybrid nanocomposite [8]. The whole device was fabricated by a simple, cost-effective and state-of-the-art printing technology. Our device was flexible, all printed, rewritable, and nonvolatile. The distinctive feature of this device was its retention time and mechanical robustness that were the highest as compared to other MoS_2_ based memory devices. Our device showed a retention time of 10^5^ s and mechanical robustness of 1500 bending cycles without any significant degradation in its characteristics. That device was bent with various diameters ranging from 50 mm to 2 mm. Furthermore, the electrode patterning technique of reverse offset for the fabrication of high-resolution RRAM devices was also reported for the first time. The characteristics of this device were far superior to most of its predecessors either based on hybrid nanocomposites or MoS_2_ RRAM devices. Mechanical flexibility of either MoS_2_ or PVA-based RRAM device was also never reported before this work. The complete fabrication process along with a schematic diagram is shown in Figure 11(b).

Fan et al. [184] have reported a similar resistive switching behavior in the hybrid nanocomposite of MoS_2_ and poly(N-vinylcarbazole) with a configuration of Au/MoS_2_-PVK/ITO. Although the memory behavior exhibited by this device was neither poor nor impressive, this was the first of its kind RRAM device as PVK was covalently bonded with MoS_2_ nanosheets to exhibit rewritable memory behavior for the first time. The effect of varying the thickness of MoS_2_-PVK active layer on the resistive switching characteristics was also explored and it was deduced that the switching ratio kept increasing with increasing film thickness with no prominent change on the threshold voltages which normally is not the case. Fan et al. also signified that annealing of MoS_2_-PVK active layer has a major effect on the resistive switching characteristics of this device including a drastic increase in the switching ratio (from 70 to 10^4^) and a considerable decrease in the SET and RESET voltages to reduce power consumption. Wang et al. [185] also studied the memory behavior in the hybrid nanocomposite of MoS_2_ and PMMA sandwiched between two layers of PMMA polymer. This study was unique in a way that the MoS_2_ was used in the form of quantum dots for the first time and their potential as a memory storage material was explored. These devices exhibited the lowest power consumption of ~2 mW when compared with other RRAM devices based on QDs of other materials including GQDS and CdSe QDs. Although switching ratio displayed by these devices was average, the SET (0.5 V) and RESET (−0.9 V) voltages were incredibly low that resulted in low power consumption. Later that year, Zhou et al. [186] reported the characteristic bipolar resistive switching behavior in the self-assembled three-dimensional microspheres of MoS_2_. The conduction mechanism in these devices was associated with the formation and rupture of filaments. Guan et al. [187] reported the resistive switching behavior in nanosheets of MoS_2_ blended with PVK polymer as reported earlier by Fan et al. with a few differences in its structure such as MoS_2_ nanosheets were thiol-modified, different top electrode, and an extra layer of PEDOT:PSS polymer. Xia et al. [188] have explored an important aspect of bipolar resistive switching behavior in the thin film of MoS_2_ by verifying the effect of changing top electrode material. This study depicted that Ag is the highly suitable material to be used as the top electrode for MoS_2_ based memory devices as compared to Ti, Cu or Al due to the easy formation of Ag filaments through the redox process taking place inside MoS_2_ thin film. The easy oxidation of Ag in MoS_2_ thin film makes it a preferred choice to be used as a top electrode. Bhattacharjee et al. [189] have reported the memory effect in the active layer of MoS_2_-PMMA nanocomposite that was a replication of the work done by our group earlier with the difference that the top and bottom electrodes were changed. The change in electrode materials had a considerable effect on the resistive switching characteristics of this already explored active layer as this time it exhibited multi-level resistive switching behavior instead of typical bipolar behavior.

**Figure 12.**
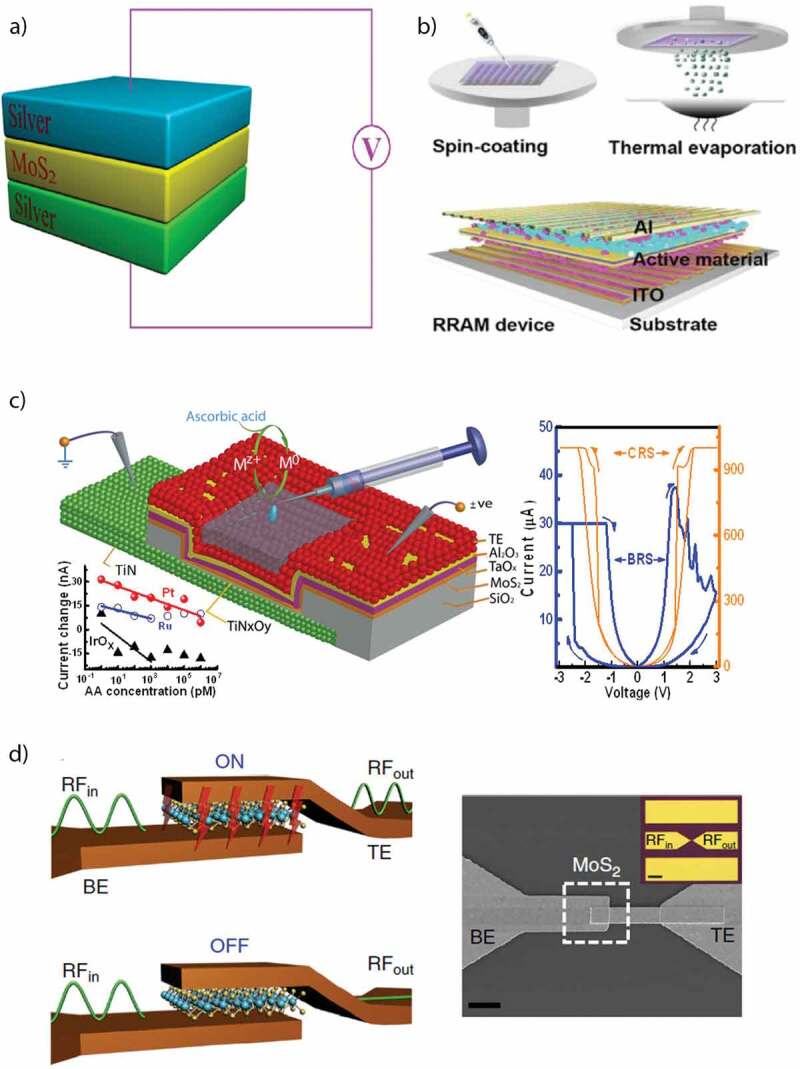
(a) Schematic structure of Ag/MoS_2_/Ag switch [181]. (b) Schematic illustration of the MoS_2_–UCNPs-based RRAM device and main fabrication process [198]. (c) Schematic view of IrO_x_/Al_2_O_3_/TaO_x_/MoS_2_/TiN RRAM structure for detection of ascorbic acid [195]. (d) Device schematics and images with material characterization. Simplified illustration of the signal transmission and device structure of the RF switches based on monolayer MoS_2_. Zoomed-in plan view SEM image of a MoS_2_ RF switch with lateral area of 1 × 1 μm^2^. Scale bar, 2 μm. The dashed box in b marks the area covered with MoS_2_. The inset is a top-view optical image of a fabricated MoS_2_ RF switch with Au electrodes. Scale bar, 50 μm [194]

Li et al. [190] investigated the switching behavior in a few layer MoS_2_-based device in the form of a lateral/planar structure instead of the conventional sandwiched structure. The main aim of this work was to suggest a method of emulating human neural network. They figured out two switching modes including conductance mediated and rectification mediated, respectively. They found out the transition from analog pulse-programmed behavior in switching to the quasi binary memory on the application of electrical stresses for the as fabricated MoS_2_ memory device. They successfully fabricated and coupled two MoS_2_ based memory cells and illustrated the potential in these devices to mimic the human brain. Chen et al. [191] proposed a memory cell with double sandwiched layer for the application of emulating neural network to mimic the human brain. In this device, MoS_2_ was used as a separating and blocking layer between two switching layers of transition metal oxides of HfO_2_ and TiO_2_. Perfect imitation of long-term penetration (LTP) and short-term plasticity (STP) is vital for successfully mimicking the neural network of human brain. The success of this device was that it managed to emulate both LTP and STP due to its volatile memory behavior owing to the role of sandwiched MoS_2_ layer that successfully blocked oxygen ions. Hou et al. [192] explored the resistive switching effect in a few layer MoS_2_-based RRAM device and simplified the three-terminal device to a two-terminal device for the ease of fabrication and simple structure. There proposed device was an important step forward to enhance the memory density. Prakash et al. [48] and Kumar et al. [193] reported the resistive switching effect in MoS_2_ based memory device with an interesting choice of bottom electrode as they used tungsten nitride (W_2_ N) and Ni-Mn-In ferromagnetic shape memory alloy, respectively, instead of conventional electrode like Pt, Au, ITO, etc. The obtained results of switching ratio, endurance and retention were of average value but this bottom electrode was a novelty in this work. Kim et al. [194] reported a breakthrough research by using the memory behavior of MoS_2_. They used its characteristics to fabricate extremely low power RF switches for the next generation internet of things (IoT) and reconfigurable communication systems. A schematic diagram of their proposed device with the characterization of used materials is shown in Figure 12(d). Simplified illustration of device structure and the signal transmission of the RF switches are also presented. Qiu et al. [195] have recently proposed an important device structure of RRAM device in which they have managed to tune the characteristics of resistive switching between bipolar, rectification and complementary resistive switching by optimizing the interfacial layer of MoS_2_. The role of MoS_2_ in this device was to provide controllable switching by confining electrons in it. Schematic diagram of the RRAM device with configuration of IrO_x_/Al_2_O_3_/TaO_x_/MoS_2_/TiN for the detection of ascorbic acid is shown in Figure 12(c). Das et al. [196] also reported a multilayer and multifunctional memory device based on MoS_2_ with the ability to store the binary data along with sensing the visible light. They grew the layer of MoS_2_ nanoparticles on the surface of bottom electrode through heating. The particle size of as synthesized MoS_2_ nanoparticles was in the range of 20 nm to 50 nm. The device showed stable and long retaining stable results with multilevel storage and light-sensing ability with a high speed. Feng et al. [197] reported a fully printed MoS_2_ based RRAM device on a flexible substrate whose schematic diagram and optical image are illustrated in Figure 12(a). They achieved a high switching ratio of 10^7^ with a distinctive feature of both volatile and nonvolatile memory behavior in a single functional layer. Zhai et al. [198] have recently reported another MoS_2_ based memory device whose complete fabrication process is illustrated in Figure 12(b). The distinctive feature of this device was its dual application, i.e. as a memory device and infrared sensor. The functional layer was based on the heterostructure of MoS_2_ nanosheets with upconversion nanoparticles (UCNPs) to form MoS_2_–NaYF_4_:Yb_3_^+^, Er_3_^+^. This novel device has its potential use in multifunctional electronic eyes and robotics. The summary of all the above discussed MoS_2_ based RRAM devices along with their typical characteristics are listed in Table 4.

**Table 4.** Summary of MoS_2_-based RRAM devices

Bottom electrode	Top electrode	Active layer	Substrate	Switching ratio	Endurance	Retention (s)	References
RGO	Al	MoS_2_-PVP	PET	10^2^	–	–	[176]
RGO	ITO	MoS_2_	SiO_2_	10^4^	–	10^3^	[177]
Ag	Ag	MoS_2_/MoO_x_	PEN	10^6^	–	10^3^	[179]
FTO	Ag	MoS_2_	Glass	10^3^	10^2^	–	[199]
Au	Au	MoS_2_	Si/SiO_2_	10^3^	–	–	[180]
Au	Au	MoS_2_	Si	10	10^3^	–	[183]
Ag	Ag	MoS_2_	Glass	–	10^2^	–	[181]
ITO	Al	MoS_2_-PVP	PET	10^3^	10^4^	10^2^	[182]
Ag	Ag	MoS_2_-PVA	PET	10^2^	10^3^	10^5^	[200]
ITO	Ag	MoS_2_	Glass	10^4^	10^2^	10^3^	[186]
ITO	Cu	MoS_2_-PMMA	PET	10^3^	10^5^	10^5^	[189]
FTO	Au	PMMA/PMMA-MoS_2_QDs/PMMA	Glass	10^2^	10^2^	10^4^	[185]
ITO	Au	MoS_2_/PVK	–	10^2^	10^2^	10^4^	[184]
ITO	Al	MoS_2_-PVK/ PEDOT:PSS	Glass	10^2^	10^2^	–	[187]
Ti	Ti/Al/Cu/Ag	MoS_2_	Si	10	–	–	[188]
TiN	Au	HfO_x_/MoS_2_/TiO_x_	–	–	–	–	[191]
ITO	Al	MoS_2_	Glass	10^2^	10^4^	10^7^	[196]
Ag	Ag	MoS_2_	Kapton	10^8^		10^5^	[197]
Cr/Au	Cr/Au	HfO_2_/Al_2_O_3_/ HfO_2_/MoS_2_	Si	10^4^	10^2^	10^3^	[192]
W_2_ N	Cu	MoS_2_	Si	10^3^	10^3^	10^3^	[48]
Ti	Ag	ZnO/MoS_2_	Ti	2	10^2^	–	[201]
Ni-Mn-In	Cu	MoS_2_	Si	10^2^	10^2^	10^3^	[193]
Ti/Au	Ti/Au	MoS_2_	Si	–	–	–	[190]
TiN	IrO_x_/Pt/Ru	Al_2_O_3_/TaO_x_/MoS_2_	–	10^5^	10^3^	–	[195]
Au	Au	MoS_2_	Glass	10^3^	20	10^4^	[194]
ITO	ITO	HfO_x_-MoS_2_-PdNPs	Glass	10^3^	10^2^	10^4^	[39]
Ti	Au	MoS_2_/PbS	Si	10^2^	10^3^	10^4^	[202]
ITO	Al	MoS_2_–UCNPs	Glass	10^4^	10^2^	10^4^	[198]

## 4. Role of other 2D materials in RRAM devices

**Figure 13.**
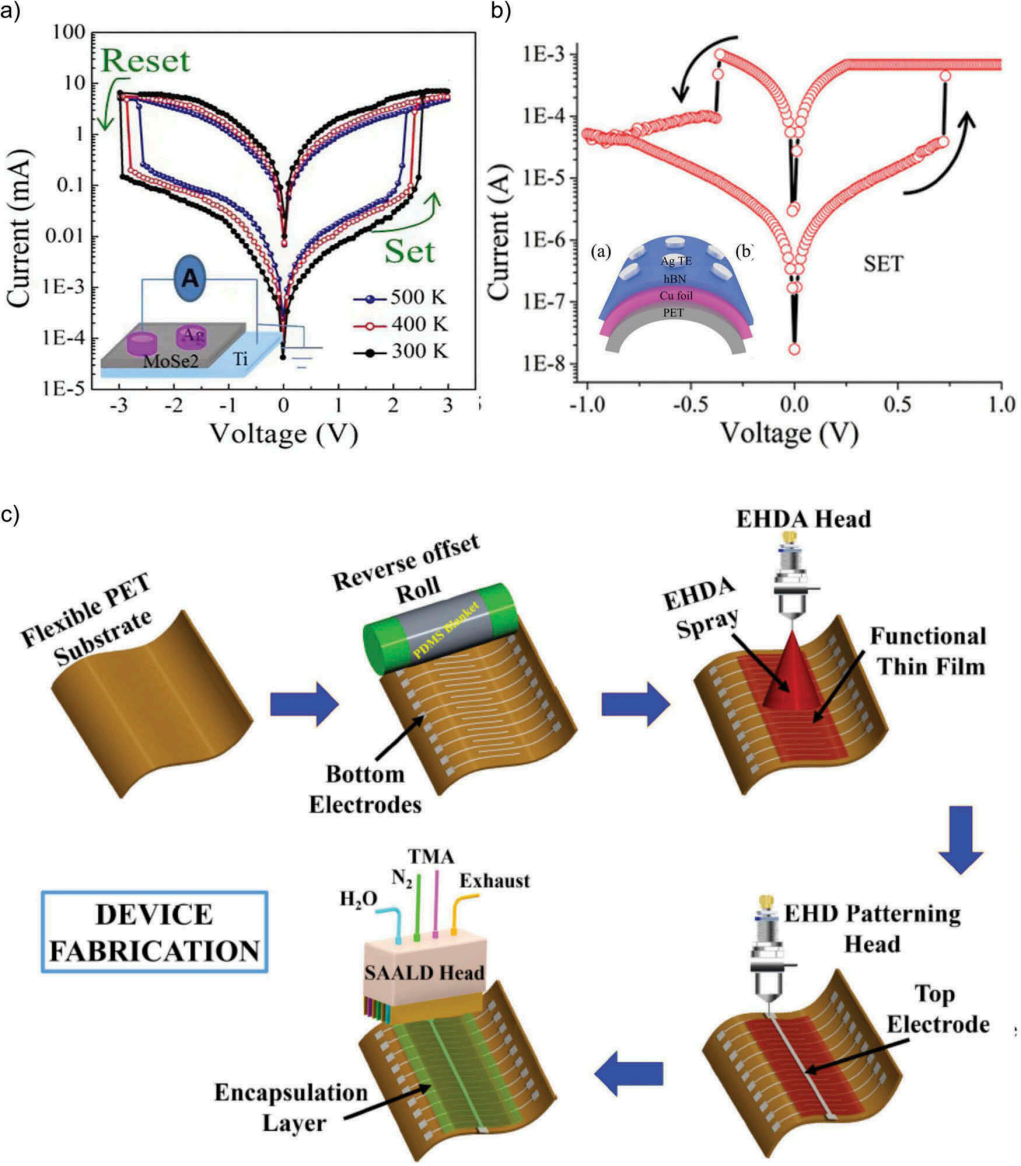
(a) The current–voltage (I–V) curves of Ag/MoSe_2_/Ti structure at 300 K, 400 K and 500 K, respectively; the inset shows the device structure [208]. (b) The switching characteristics for the hBN memory device after electroforming process with Schematic of the Ag/hBN/Cu foil on PET substrate device arrays [210]. (c) Schematic diagram of entire device fabrication through all-printing technology followed by encapsulation of atomically thin film of Al_2_O_3_ to enhance its lifetime [213]

MXenes are another important class of 2D materials being discovered whose resistive switching behavior was first explored by Yan et al. [203] when they used a single nanorod of MoSe_2_ to connect a Ag anode with an Au cathode in a planar structure device. Although the switching ratio achieved by this group was only ~50, it introduced a new potential candidate for the active layer material in the future flexible nonvolatile memory devices. Inspired by these results, Han et al. [204] studied the memory effect in the nano-islands of MoSe_2_ array embedded in a TiO_2_ matrix. The resistive switching characteristic of this RRAM device exhibited stable memory behavior in both illuminated and dark conditions. The device exhibited photo-controlled memory effect with the characteristic bipolar resistive switching behavior and its switching ratio was enhanced with increasing light intensity. Another study with a similar approach was published by Zhang et al. [205] and they reported the memory effect in the nano-islands of MoSe_2_ grown through exactly the similar technique on the same substrate with the only difference that these MoSe_2_ nano-islands were not embedded in TiO_2_ arrays. This study also did not include the effect of photoluminescence on the resistive switching characteristics of MoSe_2_. The results of this device were extremely poor with a switching ratio of only ~12. Zhou et al. [206] reported the RRAM behavior of MoSe_2_ doped ultra-long Se microwires. Although the switching ratio (~10^2^) of this MoSe_2_ based memory device was much better than the one reported by Zhang et al., both the switching ratio and retention time were insufficient. Recently, Han et al. [207] have again reported the resistive switching behavior in the hexagonal MoSe_2_ nanosheets. Han et al. explored the nonvolatile memory behavior in the nanosheets of hexagonal MoSe_2_ due to the formation of Ag filaments. Unlike the typical sandwich structure of RRAM devices, this was based on a planar structure with Ag acting as the anode and cathode. The obtained results were average with an electrical endurance of 100 cycles and a switching ratio of 70. Most recently, Li et al. [208] have reported an extremely important study in which they have explored the effect of temperature on resistive switching and magnetic properties of MoSe_2_. It was deduced from the obtained results that magnetism of MoSe_2_ nanosheets had an inverse relation with temperature while their memory behavior was regulated by temperature. The switching voltage of these devices were like those as reported by Han et al.; however, it kept on decreasing with increasing temperature, i.e. power losses in MoSe_2_ memory device can be decreased by increasing temperature. However, the switching ratio decreases with increasing temperature. A schematic diagram of their device along with its I–V characteristic curves is illustrated in Figure 13(a). Zhang et al. [51] reported the ultra-fast multi-level resistive switching effect with a synaptic behavior in the RRAM device based on molybdenum ditelluride (MoTe_2_). They achieved a fast switching speed on less than 5 ns with a better control over switching properties.

The first report on the resistive switching behavior of hBN-based RRAM device was presented by Jain et al. [209]. They explored that the highly crystalline thin film of hBN insulator exhibited unipolar resistive switching behavior as it was not dependent on the polarity of applied bias. Although Jain et al. showed the potential of data storage in hBN, they did not study its switching properties in depth and this extremely important 2D material was left high and dry for a couple of years until Qian et al. [210] reported the thorough study on the resistive switching properties of hBN by fabricating the simplest structure of RRAM device in which thin film of hBN was sandwiched between two metallic electrodes. The results illustrated in this study were important as they showed the potential of dielectric hBN as a flexible memory material. They also explored the effect of changing top electrode on the switching properties of hBN and evaluated the results of its mechanical robustness by bending it up to a radius of 7 mm. The memory behavior illustrated by this dielectric 2D material was comparable to its predecessor 2D materials such as graphene and MoS_2_. A schematic diagram of their RRAM device along with its characteristic I–V curve is shown in Figure 13(b). Inspired by the promising results of Qian et al. our research group formed a hybrid nanocomposite of hBN with PVA polymer sandwiched between ITO and Ag on a flexible PET substrate to provide highly stable, scalable and reliable characteristic bipolar resistive switching behavior [9]. Our device was fabricated through simple and cost-effective all printed technology. The obtained results of this device were superior to many other RRAM devices based on nanocomposites of PVA polymer including its mechanical robustness, switching ratio, retention time, power consumption and electrical endurance. The conduction mechanism was proved to be based on formation and rupture of metallic filaments through studying the effect of temperature and device size on the bistable resistive states (HRS/LRS). The step by step fabrication process of our proposed memory device is shown in Figure 13(c). Recently, in the year 2019, Jing et al. [211] have reported the resistive switching-based nonvolatile memory behavior in hBN-based RRAM device deposited through CVD. This was an important study as it proposed a transfer-free fabrication technique of multilayer hBN thin film on a nickel-coated wafers of silicon. The conventional techniques were expensive, slow and cracks used to appear in the thin film during the transfer process that could directly alter the electrical characteristics of the memory device. They attributed the resistive switching effect of their memory device to the percolation paths across the hBN stacks and thin layer of TiBN formed at the interface Ti/hBN.

Tungsten disulfide (WS_2_) is yet another 2D material with promising electrical and mechanical properties whose resistive switching properties were not explored until recently our group published the results exhibiting the memory behavior of WS_2_. The switching characteristics illustrated by WS_2_ were far better than many other earlier reported single 2D-material-based RRAM devices including electrical endurance and mechanical robustness. This study will encourage other researchers to explore the switching characteristics of WS_2_ based nanocomposites to further improve its switching ratio and power consumption. These devices showed impressive retention time of 10^5^ s as they were encapsulated with atomically thin film of aluminum oxide (Al_2_O_3_) to enhance their lifetime. Das et al. [212] were the second group in line after our team to propose a memory device based on the active layer of WS_2_. The thin film of WS_2_ was grown through the CVD. They achieved a switching ratio of 10^3^ with 100 endurance cycles. Recently Li et al. [53] have proposed a completely printed few layer WSe_2_-based RRAM device fabricated through aerosol jet printing technique in the year 2019. This device had an interesting characteristic as it showed both volatile and non-volatile unipolar memory behavior with a reasonable retention time of 2.5 h. The device was fabricated on a flexible Kapton tape and gave stable results up to a bending radius of 5 cm. The summary of all the above-discussed RRAM devices based on other 2D materials (hBN, MoSe_2_, MoTe_2_, WS_2_, and WSe_2_) along with their typical characteristics are listed in Table 5.

**Table 5.** Summary of other 2D-Materials (hBN, MoSe_2_, MoTe_2_, WS_2_ and WSe_2_)-based RRAM devices

Bottom electrode	Top electrode	Active layer	Substrate	Switching ratio	Endurance	Retention (s)	References
ITO	Ag	hBN/-PVOH	PET	10^2^	10^3^	–	[9]
ITO	Ag/Au/Cu	hBN	PET	10^2^	10^2^	10^3^	[210]
Ag	Ag	hBN/GQDs	PET	10^3^	10^3^	10^5^	[40]
Ag	Au	MoSe_2_	Si/SiO_2_	50	10^2^	–	[203]
FTO	Ag	MoSe_2_-TiO_2_	Glass	10^2^	50	–	[204]
FTO	Ag	MoSe_2_	Glass	5	50	–	[205]
Ag	Ag	MoSe_2_	SiO_2_	10^2^	10^2^	10^3^	[206]
Ag	Ag	MoSe_2_	Si	70	10^2^	–	[207]
Ti	Ag	MoSe_2_	Glass	10	–	–	[208]
Ti/Au	Ti/Ni	MoTe_2_	SiO_2_	10	10^2^	10^5^	[51]
Pt/Ti	Al	WS_2_	Si/SiO_2_	10^3^	10^2^	–	[212]
Ag	Ag	WSe_2_	Kapton	10^3^	–	10^4^	[53]
Ag	Ag	WS_2_	PET	10^3^	10^2^	10^5^	[213]

## 5. Multi-2D-materials-based RRAM devices

Several studies have been carried out on exploring the resistive switching characteristics of hybrid nanocomposites, i.e. blending a 2D material with other materials such as polymers however only a few reports are available in which a blend of two or more 2D materials is used as the functional layer of RRAM device. The first study on the nanocomposite of two 2D materials was performed by Yin et al. [214] who used a blend of MoS_2_ and graphene oxide as the sandwiched layer between two metallic electrodes. Although the RRAM devices based on graphene oxide showed impressive results, the motivation of this study was to lower the values of SET and RESET voltages of GO-based memory devices by forming a blend with MoS_2_ in order to reduce power consumption. The obtained results were encouraging as the conductivity of MoS_2_-graphene oxide thin film was increased that resulted in decreasing the threshold voltage up to ≤1.5 V with a stable switching ratio of 10^2^.

Shinde et al. [215] fabricated RRAM device with a different structure in which they used bilayer functional layer with the first layer of graphene and the second layer of an organic polymer (PMMA) blended with MoS_2_. This pair of MoS_2_ and graphene exhibited ten times better switching ratio as compared to the MoS_2_-graphene oxide reported earlier by Yin et al. Shin et al. [216] published an article to report the nonvolatile memory behavior in the functional thin film of MoS_2_ nanosheet embedded with graphene oxide. The obtained results were very important as the fabricated device displayed multilevel resistive switching with an added advantage of high-density data storage. Nanosheet of MoS_2_ was sandwiched between two layers of graphene oxide to exhibit at least four resistive states and average switching ratio of 10^2^. This work also verified that embedding MoS_2_ between two dielectric layers like graphene oxide makes it an excellent charge storing material. Owing to the huge potential of making the nanocomposites of 2D materials, our research group has recently explored the resistive switching behavior in the novel nanocomposite of hBN-GQDs. The device was all-printed and had a planar structure with a protective layer of atomically thin film of Al_2_O_3_ deposited by spatial atmospheric atomic layer deposition (SAALD) system. This device was fabricated on a flexible PET substrate with impressive electrical and mechanical properties such as switching ratio (10^3^), electrical endurance (10^3^ biasing cycles), retention time (10^5^ s) and mechanical robustness (2000 cycles) without any prominent decay in either resistive state (LRS/HRS).

**Figure 14.**
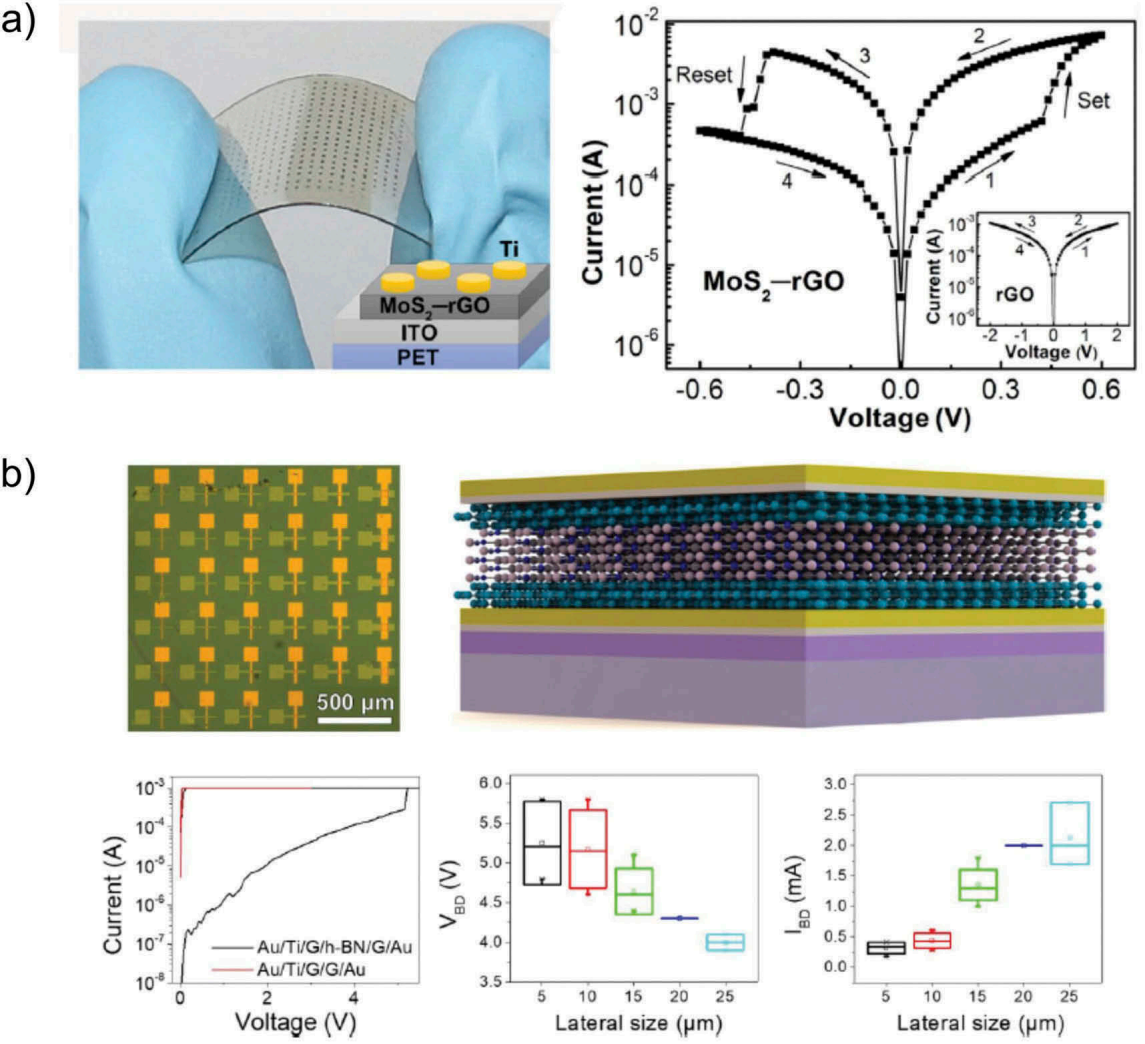
(a) Photo of the Ti/MoS2-rGO/ITO flexible memory device and schematic of the memory unit of the device. Typical I-V curve of the Ti/MoS2- rGO/ITO device plotted on a semilogarithmic scale; the inset displays the typical I-V curve of the Ti/ rGO/ITO device[45].(b) Device structure and forming process analysis. Optical microscope image of a matrix of deviceswith cross-point structure, and its cross-sectional schematic with Au/Ti/G/h-BN/G/Au/Ti/SiO2/Sistructure from top to bottom. Forming I-V curves of one fresh Au/Ti/G/h-BN/G/Au memristor (black) and a control Au/Ti/G/G/Au structure (red); the results indicate that the dielectric breakdown in related to the h- BN insulating stack. Statistical analysis of VBD and IBD shows an increasing VBD and decreasing IBD with smaller device area[46]

Recently, many research groups tried to fabricate a memory device by using two or more than to 2D materials in a single RRAM device. These groups include Zhu et al., Choudhary et al., Wu et al., and Yalagala et al. Zhu et al. [43] reported an entirely 2D material RRAM device with three stable resistive states that could be used for multiple storage of data. They sandwiched a multilayer thin film of hBN between multilayer thin films of graphene with a device configuration of Au/Ti/G/hBN/G/Au and device area of 5 μm × 5 μm. Three instead of two resistive states were realized by limiting the magnitude of compliance current. Furthermore, all the 2D materials (two layers of graphene and a single layer of hBN) used in this work were deposited through CVD instead of using the conventional technique of mechanical exfoliation. A schematic diagram along with the electrical characterization results are shown in Figure 14(b). Choudhary et al. [47] reported a RRAM device based on the combination of GO and MoS_2_ nanocomposite. The effect of changing bottom electrode on the switching characteristics of this device was also studied by using Al and ITO in two different devices. Interestingly the device with the Al bottom electrode illustrated superior RRAM behavior as compared to the ITO with a ten times better OFF/ON ratio and low values of threshold voltages for SET/RESET states. The possible reason for this difference can be attributed to the higher concentration of oxygen vacancies at the interface of active layer and Al electrode that is not present at the interface of this device with ITO as the bottom electrode. Wu et al. [217] also proposed a similar device as that of Choudhary et al. with the prominent features of multilevel storage, low operating voltage and high flexibility. They used RGO instead of GO as the composite material with MoS_2_ and their choice of electrodes combination was also distinct. They also achieved multiple resistive states in their fabricated device by controlling the magnitude of compliance current. This device showed better electrical and mechanical characteristics of a nonvolatile RRAM device. The optical image, schematic diagram and I–V characteristic curve of the proposed RRAM device are shown in Figure 14(a). Yalagala et al. [218] proposed a memory device based on the active layer of graphene and MoS_2_ with Ag and Cu as the top and bottom electrodes, respectively. They experimented the fabrication of their device on a flexible paper substrate to make their device cost effective and easily disposable. The summary of all the above-discussed RRAM devices based on other composite 2D materials along with their typical characteristics are listed in Table 6.

**Table 6.** Summary of multi-2D-materials-based RRAM devices

Bottom electrode	Top electrode	Active layer	Substrate	Switching ratio	Endurance	Retention (s)	References
ITO	Al	MoS_2_-GO	Glass	10^2^	–	–	[214]
SiO_2_	Au	Graphene/PMMA-MoS_2_	Si	10^3^	–	–	[215]
Al	Au	GO/MoS_2_/GO	SiO_2_	10^2^	10^2^	10^8^	[40]
Au	Au	Ti/Graphene/hBN/Graphene	Si/SiO_2_	10^4^	10^2^	–	[43]
Al	ITO	MoS_2_-GO	Si	10^2^	10^4^	10^4^	[47]
ITO	Ti	MoS_2_-RGO	PET	10	10^2^	10^4^	[217]
Ag	Cu	Graphene-MoS_2_	Paper	10^4^	10^2^	10^4^	[218]

## 6. Conduction mechanism of 2D-materials-based RRAMs

RRAMs change their states between HRS and LRS on the application of externally applied electric field due to change in conductivity of a material. Conduction mechanisms of 2D-materials-based RRAMs can be broadly divided into two categories including interface of electrode-dielectric (involving the formation of a filament and oxygen vacancies) and the other one dependent upon the active material itself only (SCLC, ohmic conduction, Poole-Frenkel emission, hopping conduction, and redox mechanism). Determining the exact switching mechanism of RRAMs has never been an easy task but owing to the advancements in technology, more insights into this difficult yet important problem are being explored. SCLC is the highly reported conduction mechanism in 2D-materials-based RRAMs in which charge carriers follow Ohm’s law or thermionic emission at low applied voltage but when the magnitude of applied voltage reaches the threshold voltage, more charge carriers are injected and conduction shifts from ohmic to SCL. The electrode material also plays a vital role in the conduction mechanism of RRAMs due to filament formation/migration of oxygen vacancies. Filament formation occurs when the atoms of top electrode begin to diffuse into the active layer itself whereas the SCLC mechanism is dependent upon the electrical properties (trap spacing, energy level, trap density, drift mobility, etc.) of 2D material. Growth of conductive filaments is due to the accumulation of defects such as metallic cations and oxygen vacancies that causes the device to operate in SET state while the formed filament is usually deformed through the thermal or electric effect. The conduction mechanism based on filament formation and rupture can be divided into three categories, namely, valence change mechanism (VCM), electrochemical metallization mechanism (ECM) and thermochemical mechanism (TCM) whose details have been discussed by Li et al. [219]. Diameter (size) and quantity of conductive filaments determine the performance of RRAM device. Uncontrolled filament growth can result in poor performance such as low stability, low endurance and short lifetime. Efforts must be done to control the growth and rupture of filament growth. This process can somehow be controlled through doping the resistive switching layer of 2D material and by inserting another layer.

The switching mechanism of 2D-materials-based RRAMs can also be further classified in terms of polarity of applied voltage, i.e. bipolar switching and unipolar switching. In bipolar type switching, SET and RESET process occur at two different polarities whereas in unipolar switching, both SET and RESET process can occur at either (positive or negative) polarities. In unipolar mechanism, the switching between bistable resistive states occurs due to the magnitude of applied voltage. Bipolar and unipolar switching mechanism can coexist in a single RRAM device, such RRAMs are said to have nonpolar switching mechanism. From the above discussion, it can be deduced that the conduction mechanism of a RRAM device based on 2D materials is a very sensitive topic that can be affected by the choice of electrode material, fabrication technology, film thickness of the 2D material, etc.

## 7. Challenges and future prospects

A few of the RRAM devices based on graphene registered record-breaking endurance value while other 2D-material-based devices illustrated good switching speeds and low operating voltages (<1 V). These 2D-materials-based RRAM devices have shown a lot of promise in only a decade of research behind them as compared to more than six decades of research work on TMO-based RRAM devices that is an encouraging sign for the researchers to keep working in this area. Among the other factors that need to be improved in near future for the development of 2D materials-based RRAMs, the fabrication technology is one of the key areas. For instance, one of the best endurance values achieved in a non-carbon 2D material-based RRAM device (10^3^ cycles in Ag/MoS_2_/Ag) was fabricated by using Ag paint as the top electrode and Ag foil as bottom electrode with a cell size of ≈ 0.1 mm^2^. These fabrication methods are not suitable for industry therefore, the future work in 2D-material-based RRAMs should focus on the development of new fabrication techniques that are highly compatible with industry. Annealing treatments after transfer process to eradicate remains of polymer may also be an option. Consequently, transfer process should be eluded whenever possible, not only because of the problems of device performance, but also because it elongates the fabrication process hence resulting in higher fabrication cost. The best result would be to mature transfer-free methods, but the direct growth of graphene-related materials on transition metal oxides is a long-term objective that will take further time for realization. The use of insulating layered materials as resistive switching medium is desired because, primarily, they do not need to be transferred, and, secondly, the lack of cracks can be verified by the observation of a forming set process. Recent research works on RRAM devices based on layered materials have reported CVD-grown hBN that does not require any transfer step with a deficiency that they also used metallic foils. When using hBN as a resistive switching medium, it becomes possible to avoid the standard transfer process, and the catalyst substrate for the CVD growth can be used as a bottom electrode. The direct growth of graphene-related materials by CVD on flat wafers coated with metal is highly desirable. A different option is to use LPE graphene-related materials insulators that can be spin-coated on random substrates, but they might present inconsistency, given their large unevenness (classically ≈20 nm), much more than flat graphene-related materials prepared by CVD. The use of coating techniques that could decrease the irregularities below 1 nm is compulsory. Note that the irregularities of transition metal oxides for RRAMs (typically grown by ALD) are ≈0.2 nm. Future work on RRAM devices based on 2D materials should be aimed at reducing the device size (<1 μm^2^), emphasis on representing device performance (i.e. retention, endurance, power consumption, and switching time) above the requirements of NVM technology and contain analyses of variability and reliability.

The second challenge related to transferred single-layer graphene as interface electrode RRAMs is their potential to be further reduced in size. The major disadvantage faced by these devices due to large size is that several cracks begin to appear in single-layer graphene mostly after being transferred. These cracks in turn become the reason for the formation of conductive filaments in large size memory cells based on single-layer graphene that play undesired role in the conduction mechanism. Since the retention and endurance times are related to the conductive filament properties, the presence of cracks and their influence cannot be neglected. Future endeavors should be aiming to make sure that no cracks are present in the transferred single-layer graphene electrodes. Other than reducing the cell size, another alternative is the use of MLG, which has fewer cracks and is more resilient towards mechanical fractures during transfer process. Several graphene-related-materials-based RRAM devices have been fabricated by polymer-scaffold-assisted transfer method that did not assess the occurrence of residues on the surface of graphene-related materials after removing the polymer. These might diminish the size of device, given their insulating nature and higher thickness (>10 nm). This process is arbitrary, but can be concentrated by using superior cleaning techniques, that might result in device-to-device inconsistency. The third important challenge being faced by the 2D-materials-based RRAMs lies in the fact that how excellent device performance can be achieved through engineering device structure, i.e. either by varying the electrode or the functional layer. Various device structures have already been explored including the planar structure, sandwich structure, bilayer RRAM, trilayer RRAM, using two layers of different 2D materials, using 2D material with a polymer, etc. This is still an open area for the researcher working in the field of 2D-materials-based RRAMs.

The fourth significant area open for the researchers to contribute is to work on further understanding the electrical switching behaviors and conduction mechanism of 2D-materials-based RRAMs that have been rarely explored. It is vital to carefully engineer the design of the as developed memory devices based on 2D materials to attain their high-performance in future. It should be well known that several aspects are imperative in the realization of high-performance NVM devices based on 2D nanomaterials. For instance, the quantity of functional groups on 2D nanosheets (e.g. GO) might affect the device performance.

The fifth aspect that can be further explored for improving existing 2D-materials-based RRAMs is to focus on the characterization techniques to characterize nanoscale surfaces, such as topographic AFM maps. Researchers have successfully reported the formation and rupture of nano-filaments from one electrode to the other electrode through the active layer and verified their theoretical analysis by using high-resolution TEM images. The sixth contribution in the field of 2D-materials-based RRAMs can be the use of atomistic modeling and simulations to explain the tentative observations. Only a little work has been done on simulating the results of 2D materials-based RRAMs. Software like COMSOL can be used for simulating the results, varying the values of different variables and defining the boundary conditions of 2D RRAMs. Simulating the results of such devices will be helpful in predicting the potential of certain materials and device structure before directly starting experiments which will in turn save time and materials. Furthermore, researchers can easily vary the values of different parameters like device size, film thickness, etc. to observe their effect on the switching characteristics of the device. The seventh area of research related to 2D-materials-based RRAMs is to fabricate them on flexible substrates due to their characteristic of immense mechanical endurance. This distinctive characteristic of 2D-materials-based RRAM devices will pave a new way for the development of next-generation flexible NVM RRAM device. A summary of flexible 2D-materials-based RRAM devices has been compiled in Table 7.

**Table 7.** Summary of 2D-materials-based devices fabricated on a flexible substrate; hRGO and lRGO stand for highly reduced and lightly reduced graphene oxide, respectively

Device	Substrate	Radius	Mechanical	Electrical	Ratio	References
Al/PMMA/graphene/PMMA/ITO	PET	10 mm	–	10^5^	10^7^	[83]
SLG/Al_2_O_3_/HfO_x_/Al_2_O_3_/ITO	PEN	5 mm	–	–	–	[90]
ITO/G-PVP/Ag	PET	15 mm	–	40	35	[120]
G/CNT/Al_2_O_3_	PET	8 mm	10^3^	10^2^	10^3^	[60]
MLG/PMMA/Al	PET	4.2 mm	10^4^	–	10^6^	[61]
G/P3BT:PMMA/Al	PET	10 mm	10^7^	10^7^	10^6^	[64]
G/TiO_2_/Pt	PEN	10 mm	10^2^	–	10^2^	[65]
Al/GO/Al	PES	9 mm	10^3^	–	10^2^	[148]
Al/GO/ITO	PET	4 mm	10^2^	10^2^	10^3^	[220]
hRGO/lRGO/hRGO	PET	5 mm	10^3^	–	10^3^	[124]
ITO/GO/Ag	PET	–	–	–	5	[155]
PEDOT:PSS/PVP/APTES/RGO/ PVP/pentacene/Au	PES	–	–	10^2^	–	[131]
ITO/GO/Al	PET	–	–	10^2^	10^2^	[149]
Ag/Al_2_O_3_/RGO/PMMA/Pentacene/Au	PET	5 mm	10^2^	10^2^	10^4^	[125]
ITO/ZnO/GO/Al	PET	4 mm	10^3^	10^2^	10^2^	[163]
Al/GO/Cu	PES	4 mm	–	90	10^4^	[221]
ITO/Au NPs-RGO-PVA/Al	PET	–	50	–	10^3^	[165]
ITO/GO/ITO	PES	–	–	–	10	[170]
ITO/PVK:GO/Al	PET	–	–	–	10^2^	[173]
Pt/GO/Ti/Pt	PEN	2 mm	10^3^	10^2^	10^4^	[174]
ITO/hBN-PVOH/Ag	PET	4 mm	10^2^	10^3^	10^2^	[9]
Cu/hBN/Ag	PET	7 mm	10^2^	10^2^	10^2^	[210]
Ag/GQDshBN/Ag	PET	2 mm	10^2^	10^3^	10^3^	[40]
RGO/MoS_2_-PVP/Al	PET	–	–	–	10^2^	[176]
Al/2 H-MoS_2_ -PVP/ITO	PET	–	–	10^4^	10^2^	[182]
Ag/MoS_2_-PVA/Ag	PET	2 mm	10^2^	10^3^	10^2^	[8]
ITO/PMMA-MoS_2_/Cu	PET	–	10^2^	10^5^	10^3^	[189]
Ag/WS_2_/Ag	PET	5 mm	10^2^	10^2^	10^3^	[213]
G/MAPbl_3_/Au	PET	4 mm	10^3^	10^2^	50	[73]
ITO/MgO-PVP-G/Ag	PET	–	10^2^	10^2^	10^3^	[44]
Ag/WSe_2_/Ag	PEN	5 cm	–	–	10^3^	[53]
ITO/MoS_2_-RGO/Ti	PET	10 mm	–	10^2^	15	[217]
Cu/GO-MoS_2_/Ag	Paper	–	10^3^	10^2^	10^4^	[218]

The future directions for the researchers working in 2D materials-based RRAM devices can be summarized as below:

1. Fabrication technology for the development of 2D materials-based RRAM device will see major improvements, especially in the synthesis of 2D materials and eliminationof the transferring step involved in majority of such devices. The fabrication technology should be quick, cheap and have a high throughput and above all, it should be compatible with industry.
2. Reducing the number of cracks to enhance the electrical characteristics of 2D materials-based RRAM devices as these cracks are responsible for the formation of conductive filaments. The electrical characteristics such as endurance, retention time and switching ratio can be improved. There are two possible ways to reduce the number of cracks, i.e. by reducing the device size and by using multilayers of 2D materials instead of single layers.
3. Device structure of 2D materials-based RRAM devices still needs to be finalized including the selection of number of layers, sandwich or planar structure and pair of materials to be used in those layers.
4. Various conduction mechanisms of 2D-materials-based RRAM devices have been proposed in the last decade; however, likewise other RRAMs, this particular aspect needs to be further explored for the better understanding of this field.
5. Advancement in the characterization techniques to characterize nanoscale surfaces is needed.
6. Simulations of 2D materials-based RRAM devices before experimentally fabricating them will help in saving materials, reducing the cost of fabrication and predict the effect of varying various parameters such as device size, material composition, environmental conditions, etc.
7. One of the major advantages of 2D materials-based RRAM devices over other RRAMs is the mechanical endurance of 2D materials due to which they can be used for the fabrication of flexible devices. The future of electronics is heading towards flexible devices so researchers should try to find a way in which flexibility can be introduced in all 2D materials-based RRAMs without compromising their characteristics.

## 8. Conclusions

In this review paper, we have thoroughly compiled and discussed the advancements in the RRAM devices based on 2D materials over the last decade and compared their performance with each other as well as with RRAMs based on other materials. These 2D materials include graphene, GO, hBN, MoS_2_, WS_2_, MoSe_2_, and composites of 2D materials. The contribution from various researchers working in this area has been thoroughly discussed by highlighting their strengths and weaknesses. After the in depth analysis of 2D-materials-based RRAMs over the last decade, we can conclude that the unique characteristics of these ultrathin, layered materials have proved to be helpful in fulfilling the highly desired requirements of flexible, nonvolatile, reliable and transparent memory device of next generation. Though numerous RRAM devices based on 2D materials have been reported to date but still there is more room left for improvement in order to finally commercialize these devices.

Graphene is the most commonly used layered material in the fabrication of 2D-materials-based RRAM devices over the last decade. They have been presented in the structure of RRAMs with the aims of i) increasing their performance as a nonvolatile memory (switching time, operating voltages, retention, endurance, power consumption) and ii) provide added abilities (transparency, heat dissipation, flexibility, chemical stability). Graphene can be used either as an electrode material in RRAMs to provide transparency and flexibility or as an interface layer between two metallic electrodes to function as a resistive switching medium in order to diminish the cycle-to-cycle variability, by eluding atomic diffusion between insulator and electrode. This can decrease the power consumption due to its low contact resistance as compared to the metallic electrodes. Moreover, it also suppresses the surface effects by eluding photodesorption and chemisorption. RRAMs based on graphene-related materials have displayed reproducible bipolar and unipolar resistive switching behavior with low operating voltages <1 V, high switching ratio >10^5^, and fast switching speed. In majority of reports, the switching mechanism is endorsed to the formation/disruption of conductive filaments in the resistive switching medium, and the atomic reorganizations in each state change are linked to the movement of inherent species and diffusion of metallic ions from neighboring layers, displaying parallelism with RRAMs based on transition metal oxides. Graphene related materials have also been embedded with nanoparticles, polymers, quantum dots, and nanorods in order to increase the performance (primarily endurance and retention). The drawback of graphene is that it is typically formed by the complicated process of CVD and inserted in RRAMs by polymer-assisted transfer process whereas BP and GO are typically formed by relatively easier methods of spin coating and LPE on a conductive wafer that functions as a bottom electrode.

The second most explored 2D material for the fabrication of RRAMs is MoS_2_ in various forms (flakes, nanoparticles, quantum dots, etc.). The remaining 2D materials (hBN, WS_2_, MoSe_2_, WSe_2_) based RRAM devices have also been recently explored as the potential active layer of the flexible nonvolatile RRAM. Based on the obtained results, it can be deduced that even by using the identical active layer of 2D material, the memory characteristics such as switching ratio, threshold voltage, electrical endurance and retention time are dissimilar from each other which implies that there must be other controlling parameters involved in affecting the device characteristics such as varying the thickness of functional thin films and electrodes. Furthermore, the switching mechanism of a memory device can also be controlled by altering the ratio of blended materials in the active layers based on composites of 2D materials. For instance, an active layer formed by blending the 2D material with a polymer is greatly affected by the concentration of 2D material. Moreover, the geometry/shape in which the same 2D material is used in the RRAM device also affects its properties so that nanorods, quantum dots, flakes and nanoribbons of the same 2D material have different memory characteristics. Another important parameter that has a huge impact on the memory characteristics of such devices is the number of layers of that particular 2D material that has to be used as the active layer. Thus, organized research efforts are required to improve the impact of all these factors on the overall device performance, including the hybridization with other materials of the active layers, material of electrodes, thickness of memory cell, surface functional groups, effect of environmental conditions (temperature and humidity), etc. It can be concluded that graphene is the best 2D material for RRAM devices that are meant to be used at extremely high and low temperatures. Graphene is also the best choice among 2D materials that can be used as an electrode of a RRAM device. Graphene has a major drawback that it is mostly produced by CVD; however, if the use of CVD is to be avoided, hBN is the best choice among 2D materials as the standard fabrication method can be avoided in this case. On the other hand, TMDs are useful if a flexible range of bandgaps and tunable oxidation states are needed in RRAMs. Although the most commonly used TMD, i.e. MoS_2_ can not match the resistive switching characteristics of graphene MoS_2_ can be used by chemical functionalization or through dissolving it with other polymers. Graphene-based RRAMs should be avoided to reduce the cost and speed up the fabrication process. A few of the research groups have also reported the optical behavior of 2D material-based RRAMs in which MoS_2_ is a better choice as compared to graphene as it has a direct bandgap of about 2.5 eV. On the other hand, graphene has a much higher thermal conductivity as compared to other layered materials that makes it a suitable choice for temperature RRAM devices whose conduction mechanism mainly depends on energizing the electrons through heat energy. In order to achieve the best mechanical properties of a 2D material-based RRAM, a heterostructure of graphene and MoS_2_ can be formed that will complement the mechanical constraints of each other as graphene has a large Young’s modulus and large yield stress whereas MoS_2_ has a higher bending modulus.

It took more than five decades of extensive research endeavors for TMO-based RRAMs to achieve high processing speeds, decent endurance, low power consumption, small size, high integration capacity and long data retention times; however, RRAMs based on 2D materials have already come close to these devices within a single decade. Despite all determinations, nonvolatile memories technological requirements like long data retention >10 years and high endurance >10^9^ cycles still stay a challenge. From the standpoint of flexibility, graphene-related materials-based RRAM devices can hold resistive switching behavior under more than >10^5^ bending stresses with radius as low as to few mm. Most studies based on resistive switching devices fabricated by using 2D materials are focused on proof-of-concept illustrations using large area (>2000 μm^2^) devices, that makes it tough to infer to real ultra-scaled RRAM devices but the remunerations of other 2D material properties (such as thermal heat dissipation and high chemical stability) on the performance of RRAM devices have not been explored yet. Furthermore, research must also be done on the simulations of 2D-materials-based RRAM devices.
